# Hydrogen Sulfide (H_2_S) Removal via MOFs

**DOI:** 10.3390/ma13163640

**Published:** 2020-08-17

**Authors:** Amvrosios G. Georgiadis, Nikolaos Charisiou, Ioannis V. Yentekakis, Maria A. Goula

**Affiliations:** 1 Laboratory of Alternative Fuels and Environmental Catalysis (LAFEC), Department of Chemical Engineering, University of Western Macedonia, GR-50100 Koila, Greece; amvro23@gmail.com (A.G.G.); ncharis@teiwm.gr (N.C.); 2 Laboratory of Physical Chemistry & Chemical Processes, School of Environmental Engineering, Technical University of Crete, GR-73100 Chania, Greece; yyentek@isc.tuc.gr

**Keywords:** H_2_S removal, MOFs, gas separation, isoreticular principle, host-guest interactions

## Abstract

The removal of the environmentally toxic and corrosive hydrogen sulfide (H_2_S) from gas streams with varying overall pressure and H_2_S concentration is a long-standing challenge faced by the oil and gas industries. The present work focuses on H_2_S capture using a relatively new type of material, namely metal-organic frameworks (MOFs), in an effort to shed light on their potential as adsorbents in the field of gas storage and separation. MOFs hold great promise as they make possible the design of structures from organic and inorganic units, but also as they have provided an answer to a long-term challenging objective, i.e., how to design extended structures of materials. Moreover, in designing MOFs, one may functionalize the organic units and thus, in essence, create pores with different functionalities, and also to expand the pores in order to increase pore openings. The work presented herein provides a detailed discussion, by thoroughly combining the existing literature on new developments in MOFs for H_2_S removal, and tries to provide insight into new areas for further research.

## 1. Introduction

Hydrogen sulfide (H_2_S) is a colorless, corrosive, water-soluble, highly toxic, and flammable acid gas with the characteristic foul odor of rotten eggs, which can be typically found in natural gas, petroleum, and biogas (a byproduct of anaerobic decomposition) [1,2,3,4]. Its separation has significant economic and environmental repercussions for the relevant industries. In this regard, different physicochemical methods have been developed and commercially adopted, such as biological treatment, chemical oxidation, chemical scrubbing, etc. Among these methods, the dry adsorption process has received the most attention for both large- and small-scale applications due to its superior performance in terms of H_2_S removal efficiency even at low temperatures and pressures [5,6].

Obviously, for any adsorption application, it is desirable to design adsorbents with high selectivity towards the target molecules and high adsorption capacity [7,8]. Traditional adsorbents, such as natural and synthetic zeolites, have received considerable attention in recent years. Structurally, zeolites are microporous crystalline materials formed by a combination of SiO_4_ and AlO_4_ˉ or exclusively SiO_4_ tetrahedra sharing a vertex, and they have been widely used in the petrochemical industry as catalytic materials and adsorbents as well as water softeners in detergents [9].

Karge et al. [10], who pioneered the studies of the properties of faujasite-type (FAU-type) zeolites, found that, on NaX zeolites, OHˉ and HSˉ groups are produced on the surface of the sorbent as a result of the dissociative adsorption of H_2_S molecules, indicating that these materials can be considered as good candidates for H_2_S removal. Moreover, there is a considerable amount of studies probing the Claus reaction between H_2_S and SO_2_ to produce elemental sulfur in cation-exchanged zeolites [11]. For example, Liu et al. [12] investigated a 4A zeolite synthesized from attapulgite to remove H_2_S from different industrial gases. The results revealed a maximum H_2_S capture capacity of 8.36 mg g^−1^ at 50 °C. Nevertheless, it is common knowledge that, although most zeolites can effectively adsorb acid species, such as H_2_S, they require energetically demanding regeneration processes (typically above 450 °C). A different method for the reactivation of these solids is chemical treatment with hydrogen peroxide (H_2_O_2_), which also poses risks as it can damage the pores of the sorbent.

Another group of solid materials that have been thoroughly examined for desulfurization processes are metal oxides. Of note is the work or Westmoreland et al. [13], who reported that Fe, Zn, Mo, Mn, V, Ca, Sr, Ba, Co, Cu, and W-containing metal oxides demonstrated thermodynamic feasibility for high-temperature desulfurization. However, high working temperatures result in loss of stability of the metal oxides [14,15] while at lower temperatures they experience poor capture performance [16]. To avoid these limitations, mixed metal Zn-based oxides came to prominence for low temperature H_2_S removal [17].

Generally, the major shortcoming for this class of materials is the irreversible structure transformation caused by the chemisorption of H_2_S, preventing their reactivation [18]. In addition, their strong interactions with H_2_S can stimulate the formation of other environmental contaminants, as is the case of Fe_2_O_3_ used for H_2_S capture_,_ which produces SO_2_ by the exhaustive oxidation of H_2_S and elemental sulfur [19]. Metal oxides also exhibit poor porosity and low surface areas (SSAs) which can also afflict their adsorption capacity. One strategy to enhance their gas capture performances and decrease the operating temperatures is to support these solids on a porous matrix, such as stable mesoporous materials (i.e., MCM-41 and SBA−15). Thus, this method introduces new perspectives to consider in order to accomplish sufficient gas removal although, in most cases, increases the implementation costs [20]. Another way is the use of metal hydroxide [21] or oxohydroxide [22] composites, which offer the opportunity of operating in lower temperatures in comparison to those of pure metal oxides, but with average adsorption capacities. It should be stressed that, unlike Fe oxides, where increased temperatures are needed to provide sufficient gas capture performance, techniques based on metal oxides are yet to be commercialized for desulfurization processes. These technical limitations can also occur in the case of other H_2_S adsorbents such as activated carbons, amino silanes, and ionic liquids [23,24,25].

That said, a need of developing holistic technologies toward the design of materials that meet the industrial requirements and conform with environmental laws and regulation standards as well as exhibit improved gas capture performance arises.

A class of inorganic-organic hybrid materials, known as metal-organic frameworks (MOFs), with a crystalline nature that bear an open and porous framework have shown decent performance in CO_2_ capture over the last twenty years, and recently they have been probed in the adsorption of acid gases (i.e., H_2_S), showing promising potential [26]. They consist of metal ions or clusters of metal ions, inorganic secondary building units (ISBUs), linked together by organic linkers. The organic units which are negatively charged molecules, are usually ditopic or polytopic organic carboxylates which, when linked to metal ions, form nodes that bind the arms of the linkers, resulting in architecturally robust crystalline MOF structures [27,28].

MOFs can exhibit significant advantages in gas selectivity and separation due to their large surface area (typically ranging from 1000 to 10,000 m^2^/g), diverse structure, molecular dimensions, and tunable functionality [29]. For instance, the chemistry of their pores can be improved by different methods, such as metal ion exchange. The ability to vary their size and nature, without changing their underlying topology and tailorability in terms of chemical functionality, affords control over the structure and properties required for given applications. MOFs can operate through physical or weak chemical adsorption interactions, thereby requiring lower regeneration energy in comparison to other methods (e.g., aqueous amine solutions) due to their lower heat of adsorption and heat capacities [30,31]. Another advantage of MOFs is that they can cover the full pore size gap between zeolites (microporous) and mesoporous silicas due to their highly tunable pores (0–3 nm up to 9.8 nm).

For most of the twentieth century, the synthesis of crystalline structures was rather fortuitous and the construction of materials with atomic precision posed an insuperable challenge. However, in 1959, Yukio and his co-workers, in an attempt to build large structures from well-defined building units, studied the crystal structure of bis(adiponitrilo)copper(I) nitrate [Cu(NC–CH_2_–CH_2_–CH_2_–CH_2_–CN)_2_]NO_3_. Using two-dimensional Fourier methods, the authors demonstrated that bis(adiponitrilo)copper(I) nitrate is orthorhombic and consists of infinite three-dimensional networks of complex ions of $[\mathrm{Cu}{\left(\mathrm{NC}–{\mathrm{CH}}_{2}–{\mathrm{CH}}_{2}–{\mathrm{CH}}_{2}–{\mathrm{CH}}_{2}–\mathrm{CN}\right)}_{2}]{\;}_{n}^{n+}$ and nitrate ions. They also showed that the copper ions were linked together by rod-like links of adiponitrile, creating a framework that was based on the widely known dense structure of diamonds, except here, due to the length of the units, the structure was open. Nevertheless, in this particular case, the openness of the structure resulted in interpenetration, thus preventing the space from being accessible [32].

The work of Yukio et al. [32] was followed by numerous efforts that were reported in the literature, but the term “metal-organic framework” itself was introduced for the first time in 1994, in a pioneering study carried out by Yaghi et al. [33]. For this work, Omar Yaghi earned the moniker “the father of MOFs”. Two additional key works in the development of MOFs were reported in 1999 [34,35]. Firstly, Williams et al. [34] synthesized a highly porous open-framework metal coordination polymer, referred as Cu-BTC (also stands for HKUST−1 and MOF−199), which was comprised of copper-based clusters and benzene tricarboxylate linkers. Not to be outdone, a few months later, Yaghi et al. [35] reported MOF-5, a structure comprised of zinc-based clusters and benzene dicarboxylate linkers. The authors showed that compared to conventionally used microporous inorganic materials such as zeolites, these robust materials exhibited considerably higher SSAs and pore volumes.

The ability to alter the metrics of MOF structures, by using an expanded version of the parent organic linker, without changing their underlying topology, gave rise to the isoreticular (having the same network topology) principle and its application in making MOFs with even larger pore apertures and lower densities (reaching up to 98 Å and 0.13 g cm^−3^) [35]. Similar to the ISBUs, these organic linkers, appropriately functionalized, can also redound in promoting the selectivity towards target molecules [36,37,38].

Intense efforts have been carried out to exploit the properties of MOFs in a large number of applications and in diverse fields, such as gas storage and separation, liquid separation and purification, electrochemical energy storage, catalysis, and sensing. However, despite the recent momentum, MOFs have yet to find a commercial impact, and concerns in terms of their cost-effectiveness and stability remain. For example, the capture of acidic H_2_S has proven to be a tough challenge, largely owing to the formation of a strong and occasionally irreversible bond. This bond can cleave other coordination bonds between the metal centers and the linkers, leading to a structural disintegration of the MOF. Moreover, in the presence of water, the formation of acidic species can accelerate their structural degradation. In particular, the H_2_S/H_2_O reaction can result in the formation of HSˉ and H_3_Oˉ species [39]. Consequently, it is important to regulate the host–guest binding interactions between MOFs and H_2_S. As a rule, in order to achieve a reversible process, these interactions should transpire through non-covalent bonding between H_2_S and the functionalized linkers, such as hydrogen bonds, or by means of donor-acceptor bonds with the uncoordinated metal sites within the structure of the MOF [40]. To predict these basal interactions density functional theory (DFT) and grand canonical Monte Carlo simulations (GCMC) have been widely implemented by numerous works, as will be discussed throughout the text below. Although the topic of desulfurization using MOFs has been thoroughly reviewed in the past [41,42,43,44,45,46,47,48], the work presented herein is the first to collect and critically review all works found in the literature that focus exclusively on H_2_S capture. As such, attention has been paid to the structural characteristics of these materials that provide significant advantages in H_2_S selectivity and separation, but also to issues related to the reversibility of the process. Efforts have also been made in identifying promising areas for future research.

## 2. H_2_S Capture via Materials of the Institute Lavoisier (MILs)

**Figure 1:**
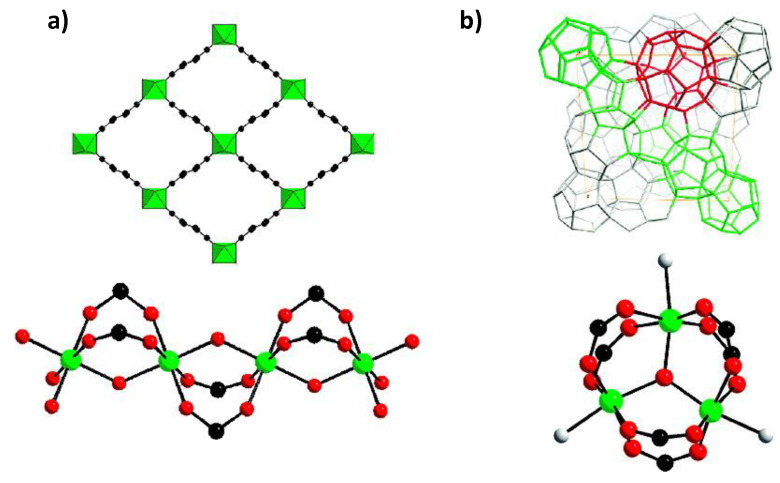
Structure of the MIL-47(V) and MIL-53(Al, Cr, Fe) solids (**a**). View of the structure of MIL−100(Cr) and MIL−101(Cr) (**b**). The corresponding inorganic subunits are shown below each structure. Metal, oxygen, and carbon atoms are shown in green, red, and black, respectively, while terminal water molecules and fluorine are shown in gray. Reproduced with permission from Ref. [49]. Copyright 2009 Journal of the American Chemical Society.

**Figure 2:**
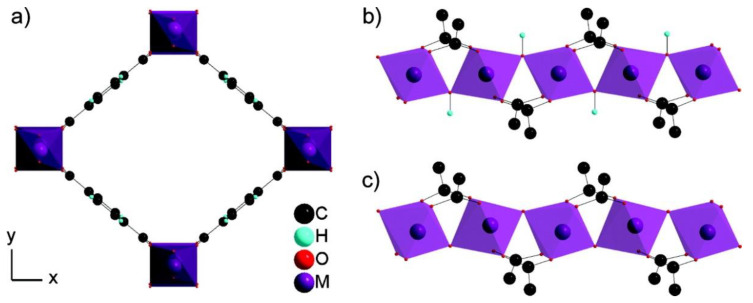
(**a**) View of the MIL-53(Cr) LP/MIL-47(V) structures along the chain (z axis), highlighting the 1D pores system with M = Cr^3+^ or V^4+^. View perpendicular to the pores of the MIL-53(Cr^3+^), (**b**) and MIL-47(V^4+^), and (**c**) with μ_2_-OH and μ_2_-O atoms linked to the Metal atom (M), respectively. Reproduced with permission from Ref. [50]. Copyright 2011 The Journal of Chemical Chemistry C.

Hamon et al. [49] pioneered the investigation of H_2_S adsorption in MOFs. In particular, the group probed several MIL-series MOFs (MIL stands for materials of the Institute Lavoisier), including the small-pore (SP) MIL-53(Al^3+^, Cr^3+^, Fe^3+^) and MIL-47(V^4+^) (approximately 11 Å), as well as, the large-pore (LP) MIL−100(Cr) and MIL−101(Cr) (25 and 30 Å cage diameter and 4.8 × 5.8 and 12.5 × 12.5 Å pore apertures respectively), as seen in Figure 1, for H_2_S capture at 30 °C. SP MILs solids, presenting the same topology but containing two different metal centers, are built from corner-sharing chains of either (M^3+^O_4_(OH)_2_) or metal oxide (M^4+^O_6_) octahedra interconnected through terephthalate moieties (SSA ≈ 1000 m^2^ g^−1^) (Figure 2). LP MILs solids consist of trimers of chromium octahedra linked with trimesate (MIL−100) or terephthalate (MIL−101) (SSA > 2000 m^2^ g^−1^). The isotherms observed for MIL-47, MIL−100(Cr), and MIL−101(Cr) MOFs were type-I-shaped, illustrating that they can be considered as rigid structures. In contrast, two step adsorption isotherms were shown for MIL-53(Al, Cr, Fe), which the authors hypothesized to be caused by the strong polarity-based interactions (hydrogen bond) between H_2_S molecules and -OH groups of the inorganic chains, resulting in pore occlusion at low gas or vapor loading.

Soon thereafter, these findings were claimed to be confirmed by the same group using a combination of IR measurements and modeling [50]. Regarding the breathing MOF (MIL-53(Cr)), isotherms were simulated to represent both the narrow and the large-pore regimes in the experimental adsorption isotherm. Even though the simulations provided a decent match with the experimental results, the analysis failed to examine the ability to predict the structural transformation. CH_4_ adsorption tests at room temperature before and after high pressure H_2_S treatment, followed by vacuum desorption (8 h at 120 °C), showed that all the small pore solids, except for MIL-53(Fe), exhibited fully recovery. The latter material completely lost its adsorption capacity, owing to the formation of iron sulfide (FeS) and the crystallization of terephthalic acid at the surface of the adsorption cell. Simply put, the structure of MIL-53(Fe) collapses and forms FeS. On the other hand, such reversibility was not observed in the mesoporous type materials, i.e., MIL−100(Cr) and MIL−101(Cr) (hydrogen bond between μ-O group and H_2_S molecules). The Henry’s constants were determined to be 72.9 mmol g^−1^ MPa^−1^ and 61.3 mmol g^−1^ MPa^−1^ for the MIL−100(Cr) and MIL−101(Cr) MOFs, respectively, while the maximum adsorbed quantities at 2 MPa were immensely high: 16.7 and 38.4 mmol g^−1^, respectively. However, their inability to regenerate hinders the use of these larger pore MIL-series (mesoporous) as potential candidates for practical applications. The authors also reported that MIL-53(Al, Cr) and MIL-47 exhibited saturation loadings of 11.8, 13.1, and 14.6 mmol g^−1^, respectively, which are considerably high in comparison to those of other conventional materials for H_2_S uptake (i.e., activated carbons: 1.8 mmol g^−1^, 13X zeolites: 5.62 mmol g^−1^).

**Figure 3:**
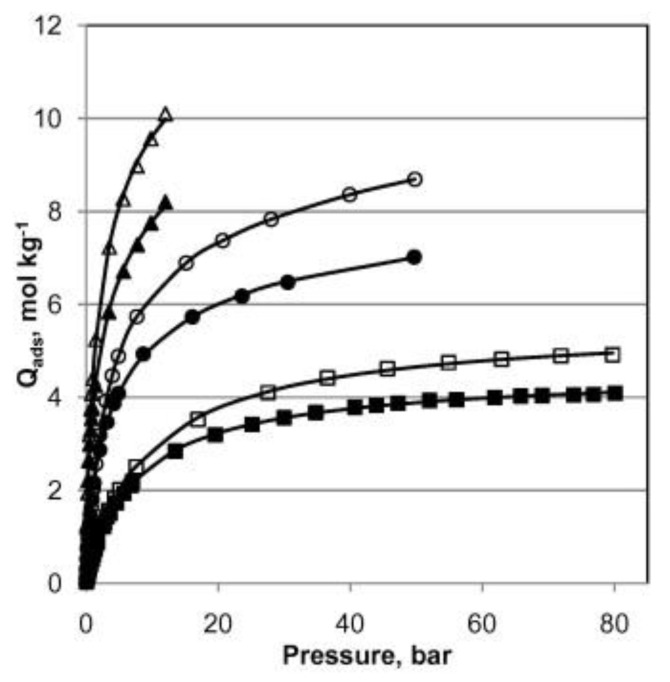
Adsorption isotherms of different adsorbates at 303.15 K on two forms of the Basolite A100: powder (empty symbols) and pellets (filled symbols). Points are the experimental data: H_2_S (triangles), CO_2_ (circles) and CH_4_ (squares); lines represent fitting curves with Toth equation. Reproduced with permission from Ref. [51]. Copyright 2012 Microporous and Mesoporous Materials.

H_2_S capture by MIL-53(Al), in both powder and pellet form, was also studied by Heymans et al. [51]. The authors conducted a thorough experimental and theoretical study of MOF-53(Al) as an adsorbent for the removal of H_2_S and CO_2_ from biogas. High-pressure pure gas isotherms of CO_2_, H_2_S and CH_4_ gave evidence of H_2_S steep uptake in comparison to those of the two other species (Figure 3). This pure compound study also revealed that MOF-53(Al) was fully regenerable at relatively low temperature (200 °C), suggesting that physisorption occurred. Alongside the simulations, the interaction of H_2_S with the framework as reported by the Hamon group was corroborated [49,50]. It was also reported that the powdered MOF-53(Al) exhibited higher H_2_S adsorption capacity, presumably owing to its increased SSA and pore volume compared to the pellet-form MOF-53(Al).

**Figure 4:**
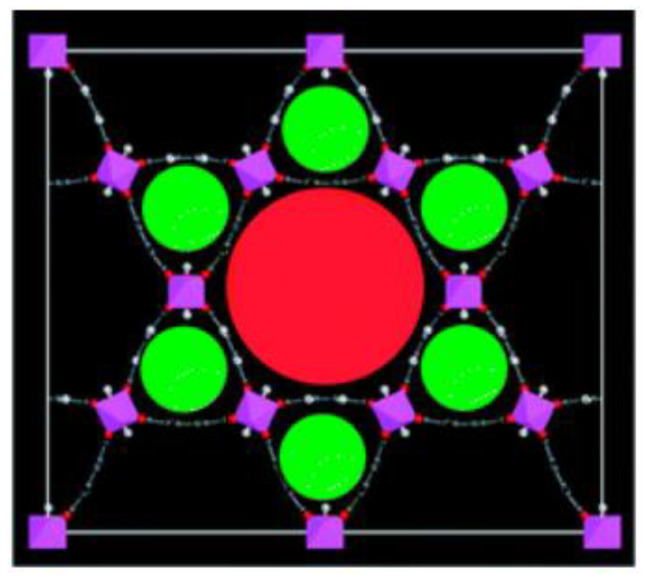
View of the crystalline structure of the MIL-68(M) (M = V, Ga, Fe, or Al) along the c axis: green and red circles denote the triangular and hexagonal channels, respectively (metal polyhedra, pink; C, gray; O, red; H, white). Reproduced with permission from Ref. [52]. Copyright 2012 Journal of Materials of Chemistry.

MIL-68(Al) was investigated at high H_2_S pressures up to 12 bar at room temperature by Yang et al. [52] using experimental and modeling (Grand Canonical Monte Carlo-GCMC) approaches. The three-dimensional networks of these materials consist of two types of channels, triangular (6.0–6.4 Å) and hexagonal (16–17 Å) running along the c axis coordinate system (Figure 4). It was reported that only a specific number of the hydroxyls were available for interaction with H_2_S, as confirmed by the simulated isotherm occurring by blocking the triangular channels, in harmony with the experimental isotherm. Moreover, the adsorption enthalpy of −21.6 kJ mol^−1^ was calculated experimentally. Bearing in mind the modeling as well as the experimental approaches, one may conclude that the triangular pores of MIL-68(Al) were blocked by some remaining organic or solvent molecules owing to the incomplete activation of the material. This partially activated material was shown to be fully regenerable for at least five consecutive cycles, without loss of capacity. Nevertheless, it is unclear from this work whether the MIL-68(Al) can resist corrosiveness of H_2_S if fully activated. However, it is also worth noting that MIL-68(Al) exhibited a rigid structure, as confirmed by its type-I-adsorption isotherm, despite the fact that it is considered as a polymorph of MIL-53(Al), which in comparison shows framework flexibility. This behavior highlights the significant role of an MOF’s framework topology in addition to the choice of the secondary building units.

**Figure 5:**
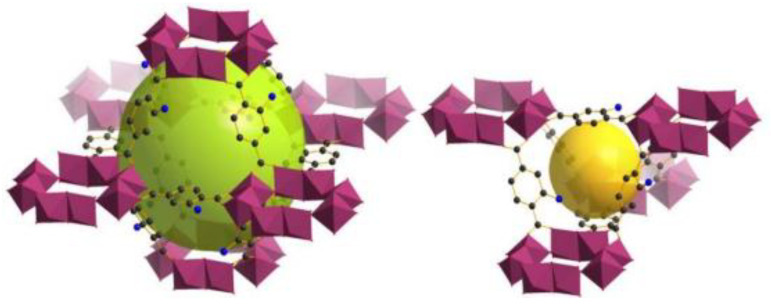
Schematic view of the cages of MIL−125(Ti)-NH_2_. Metal polyhedra, carbon, nitrogen and oxygen atoms are in violet, black, blue and red, respectively. Hydrogen atoms have been omitted for clarity. Reproduced with permission from Ref. [53]. Copyright 2013 Chemical Communications.

Vaesen et al. [53] investigated the adsorption performance of the amino-functionalized titanium terephthalate MIL−125(Ti)-NH_2_ towards its parent MIL−125(Ti) analogue, for the concurrent elimination of CO_2_ and H_2_S from biogas and natural gas using a joint experimental/modeling approach. Generally, Ti MOFs bear two intriguing characteristics, namely that they are H_2_S resistant and they can be scaled up to the multi-gram scale under ambient pressure conditions. MIL−125(Ti)-NH_2_ consists of tetrahedral (4.7 Å) and octahedral (10.7 Å) cages, accessible through triangular windows of 5–7 Å. The cage diameters of the parent material (6 Å for the tetrahedral cage and 12 Å the octahedral cage) are moderately bigger than those of the functionalized material (Figure 5). The pure-component adsorption tests at 30 °C and low pressures demonstrated reduced H_2_S adsorption capacities for both MOFs. However, the adsorption enthalpies calculations for each gas denoted that the MIL−125(Ti)-NH_2_ can achieve selective H_2_S removal over CH_4_ (∼70). In practical terms, they verified that the gas adsorption performances following H_2_S and H_2_O exposures were not compromised. Afterwards, in situ IR in the ν(H_2_S) range revealed that for NH_2_-free MOF the adsorbate/adsorbent interaction was established with the μ_2_-OH groups through μ_2_-OH·S(H_2_S) interactions (2570 cm^−1^), while for MIL−125(Ti)-NH_2_, two additional interactions occurred; when H_2_S acts as an H donor HS-H·N (from amino radical functionalization) with a 2550 cm^−1^ band, and when the adsorbate molecule acts as H acceptor H_2_S·H (NH_2_). These interactions were corroborated by GCMC simulations. Compared to other adsorbents, such as 13X zeolites (∼45 kJ mol^−1^), these amine-functionalized MOFs exhibit lower H_2_S adsorption enthalpies (∼30 kJ mol^−1^), which result in a lower energy footprint for recycling the sorbent. Moreover, the relatively high CO_2_/CH_4_ selectivity (∼7) of MIL−125(Ti)-NH_2_ allows one to envision a one-step process for the synchronous capture of CO_2_ and H_2_S. Yet still, on the grounds that these MOFs are unstable in water vapor at temperatures above 100 °C, its improvement (structural stability) is a topic for further consideration. Overall, this work corroborates the simulations carried out by Yang et al. [52], highlighting the positive role of amino functional group in capturing H_2_S molecules. 

Motivated by Biswas et al. [54], who studied the adsorption properties of CO_2_ using functionalized MIL-47-X (X = –Cl, –Br, CH_3_, –CF_3_, –OCH_3_), Sokhanvaran et al. [55] conducted molecular simulations (GCMC) to investigate the adsorption and selectivity of H_2_S, CO_2_, and CH_4_ molecules and their mixtures on the functionalized MIL-47-X (X = –OH and –OCH_3_) and pristine MOF MIL-47. The three studied MIL-47 MOFs exhibited type-I shaped isotherms, clearly indicating a rigid microporous structure. Results showed that the gas adsorption on MIL-47-X at low pressures was superior compared to that of the MIL-47. Even though the uptake at saturation decreased by functionalization, the adsorption selectivities of the MIL-47-X were higher when evaluated against the pristine MOF. In particular, using H_2_S/CH_4_, H_2_S/CO_2_, CO_2_/CH_4_ mixtures, the materials studied showed selectivity in the order of MIL-47 < MIL-47-OH < MIL-47-OCH_3._ The authors attributed this finding to the fact that the adsorption isotherms showed greater affinity for polar H_2_S over non-polar CO_2_ molecules for the materials under consideration, probably owing to the strong interaction of H_2_S with -OH groups. In combination with the results of the adsorption isotherms, the higher calculated isosteric heat of H_2_S for all MIL-47 solids compared to that of CO_2_ implied the stronger interaction between the H_2_S molecules and the atoms in the framework of MOFs. The simulated adsorption isotherm of H_2_S on the MIL-47, obtained at 30 °C, showed a decent agreement with the experimental data of the Hamon group [49].

**Figure 6:**
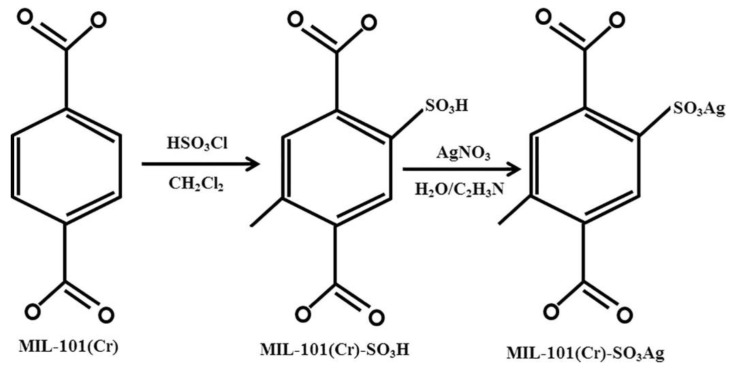
Schematic of the synthesis Nano adsorbents. Reproduced with permission from Ref. [56]. Copyright 2019 Chemical Engineering Journal.

In another recent study focusing on the MIL-family, Pourreza et al. [56] investigated the adsorption capacity of H_2_S on three different MOFs, namely MIL−101(Cr), MIL−101(Cr)-SO_3_H and MIL−101(Cr)-SO_3_Ag in a dynamic adsorption system. In particular, the MIL−101(Cr)-SO_3_H, which demonstrated high SSA, was used as a matrix to load Ag(I) by ion exchange (Figure 6). Results showed that the breakthrough time measured for the Ag-free MOFs was short while the curve was moderately steep, suggesting that these sorbents presented poor capacity in capturing H_2_S (MIL−101(Cr)—24.32 mg g^−1^/MIL−101(Cr)-SO_3_H—28.67 mg g^−1^). Conversely, the breakthrough time for MIL−101(Cr)-SO_3_Ag was longer, illustrating enhanced H_2_S adsorption capacity (96.75 mg g^−1^). Thus, although the Ag-functionalized MOF exhibited lower SSA (1534 m^2^ g^−1^) compared for example to the MIL−101(Cr) (1725 m^2^ g^−1^), its adsorption capacity was almost four times higher, indicating that sulfur molecules binded preferentially on the Ag(I) ions. Moreover, by performing 5 adsorption-desorption cycles using the MIL−101(Cr)-SO_3_Ag, the authors showed that the capacity of the sorbent remained almost unchanged (approximately 96 mg g^−1^). It was also noticed that increasing temperature had an adverse effect on H_2_S adsorption, suggesting that physical adsorption predominated. Namely, the adsorption capacity of H_2_S on MIL−101(Cr)-SO_3_Ag decreased by 25.56 mg g^−1^ following a temperature increase from 20 to 60 °C, in agreement with the exothermic nature of physical adsorption. The authors also applied the density functional theory (DFT) with a view to probe the adsorption mechanism of H_2_S on these solids and reported that the Ag-modified sorbent could double the value of the H_2_S adsorption energy compared to that of MIL−101(Cr)-SO_3_H. Simply put, the Ag atoms could improve the retention of H_2_S in the pores of the sorbent in comparison to SO_3_H-modified linker. Overall, the electrostatic forces and the amount of charge transfer terms, in conjunction with polarization, which holds a significant role in H_2_S-containing adsorption processes, could provide enhanced adsorption properties for MIL−101(Cr)-SO_3_Ag in terms of H_2_S removal.

However, serious concerns should be raised in regard to the likely future commercialization of MIL−101(Cr) due to the use of HF as modulator during its synthesis. Structurally, the nanoporous chromium terephthalate MIL−101(Cr) is composed by terephthalic acid (benzene−1,4-dicarboxylic acid), chromium salt (Cr(NO_3_)_3_·9H_2_O) and hydrofluoric acid (HF), as a modulator, in an aqueous medium. Even though HF can significantly enhance the synthesis efficacy and crystal size of this solid, it is a highly toxic and corrosive liquid capable of afflicting the nervous system. In this context, numerous attempts have been made to prepare HF-free MIL−101(Cr) sorbents.

For example, Leng et al. [57] prepared and tested a solvent-free MIL−101(Cr). Noorpoor et al. [58] prepared MIL−101(Cr) using a series of modulators (HF included) and assessed the H_2_O uptake and physical properties of products in liquids. The authors found that the sorbents synthesized by ethanoic acid exhibited enhanced SSA (i.e., 2927 m^2^ g^−1^) and H_2_O uptake (130 g g^−1^), which corresponds to 40% and 73% improvement, respectively, in comparison to the HF-containing ones. Hu et al. [59] probed highly porous HCl-assisted MIL−101(Cr). Results showed improved SSA and pore volume when evaluated against HF-assisted samples. Ren et al. [60] used methanoic acid as modulator and found relatively promising H_2_ capture for practical applications (at 1 bar and 77K the H_2_ uptake was 1.9 wt.%). Jiang et al. [61] also tested HF-free MOFs and found that these samples prepared using monocarboxylic acid as modulator presented decent SSA ranging from 2600 to 2900 m^2^ g^−1^. The study also reported increased CO_2_/N_2_ selectivity, presumably since these sorbents have the ability to tailor their particle size in a controlled manner (i.e., 19 to 84 nm). Zhao et al. [62] and Zhou et al. [63] used a combination of weak alkaline acetates, such as lithium acetate and potassium acetate, and acetic acid to obtain MIL−101(Cr) crystals and reported that the samples exhibited high SSA (3200–3500 m^2^ g^−1^) and tolerable production yield (over 60%).

Specifically, for H_2_S removal, Alivand et al. [64] conducted a very interesting work, in which they synthesized HF-free MIL−101(Cr) crystals using different HNO_3_ concentrations to study the adsorption capacity of polar and non-polar molecules (i.e., H_2_S, CH_4_, CO_2_, and N_2_) on MIL−101-HNO_3_ at different temperatures (i.e., 0, 10, 20 °C) and up to high pressures (up to 3500 kPa). Adsorption equilibrium data were well-fitted with Langmuir, Langmuir–Freundlich, and dual-site Langmuir-Freundlich models. Results showed that the MIL−101-HNO_3_−1 (HNO_3_/H_2_BDC=1) exhibited the highest SSA (3841 m^2^ g^−1^) and pore volume (1.72 cm^3^ g^−1^), this being the optimal concentration of HNO_3_. Moreover, at ambient temperature, the adsorption capacity of H_2_S, CO_2_, and CH_4_ on MIL−101-HNO_3_−1 was improved significantly (i.e., H_2_S: 21.7%, CO_2_: 29.3% and CH_4_: 12.3%) at 100 kPa in comparison to that at 3500 kPa (i.e., H_2_S: 7.1%, CO_2_: 13.7% and CH_4_: 11.5%). The authors also used ideal absorbed solution theory (IAST) to predict the selectivity of H_2_S/CH_4_, CO_2_/CH_4_, and CO_2_/N_2_ on the MIL−101-HNO_3_−1, and found it to be 12.6, 14.2, and 336.1. It is noteworthy that these IAST predicted selectivities displayed an increment of approximately 167%, 22%, and 14% at 10 kPa. Furthermore, the authors stated that the improved adsorption capacity and separation ability of HNO_3_−1-assisted sample over MIL−101-HF−1, is due to additional electrostatic adsorptive sites and more open Cr^3+^ metal sites, which were both a result of the increased SSA (3609 to 3841 m^2^ g^−1^) and pore volume (1.55 to 1.72 cm^3^ g^−1^). Finally, the authors extolled the recyclability, as well as moisture and thermal stability of MIL−101-HNO_3_−1, signaling that these materials can be regarded as superior alternatives for MIL−101(Cr) prepared by HF, in industrial desulfurization processes.

Kooti et al. [65] synthesized new hybrids consisting of nanoporous carbon (GCKP2) and MIL−101(Cr) in different ratios (i.e., ranging from 10 to 50%) and used them as adsorbents for H_2_S removal. A series of characterization methods were carried out to gain insight into the physicochemical properties of these samples. Results showed adsorption capacities of 10, 6.2, 7.9 and 6 mmol g^−1^ at approximately 90 kPa for MIL−101 samples containing 10%, 30%, and 50% of GCKP2, respectively. On the other hand, the amount of H_2_S adsorbed on pristine MIL−101(Cr) was higher, namely 10 mmol g^−1^. The authors claimed that the decreased H_2_S adsorption capacities of GCKP2-containing materials were due to their higher packing density (i.e., 1.4 times higher than MIL−101). Furthermore, the authors also obtained H_2_S adsorption equilibrium isotherms for the above samples at low pressures (up to 10 kPa) by means of Langmuir, Freundlich, and Toth models. The nonlinear behavior of the isotherms, which is most likely ascribed to the contribution of physisorption to the process, illustrates the heterogeneity of adsorption sites for hybrids that contain GCKP2.

In a recent work, Díaz-Ramírez et al. [66] reported the partial functionalization of MIL−101(Cr) with fluorine using 2,3,5,6-tetrafluoro−1,4-benzenedicarboxylate (BDC-4F) providing MIL−101(Cr)-4F(1%). Cyclic voltammetry measurements showed that the acidity of the metal centers of the modified form of the MOF was improved. The authors conducted several adsorption tests, including H_2_S adsorption at 30 °C and 15% of H_2_S volume. Results demonstrated the highest H_2_S capture for the MIL−101(Cr)-4F(1%) (36.9 mmol g^−1^) in comparison to all other mesoporous MOF materials mentioned in the literature. However, H_2_S exposure partially damaged the crystallinity in both structures.

Xu et al. [67] performed a computational study (Monte Carlo simulation) to investigate the adsorption and separation of H_2_S in the monohalogenated MIL-47-(V)-X (X = F, Cl, Br) and its parent analogue MIL-47(V). Results showed that both halogenated and initial materials exhibit a considerably high H_2_S adsorption capacity, in comparison to that of CH_4_ and N_2_. It was also noted that the adsorption isotherms of all gases in halogenated and initial MOFs are of typical type I, suggesting a rigid structure. Functionalization with halogens could improve the adsorption of H_2_S in unit volume of sorbents, notably in low-pressure range, following an order MIL-47(V) < MIL-47(V)-F < MIL-47(V)-Cl < MIL-47(V)-Br, according with the increasing polarizability of the linkers, insofar as the H_2_S mass fraction follows an opposite order MIL-47(V) > MIL-47(V)-F > MIL-47(V)-Cl > MIL-47(V)-Br, due to the rising mass density of MOFs after halogenation. The authors found that the four sorbents exhibited increased selectivity toward H_2_S molecules in H_2_S/CH_4_ and H_2_S/N_2_ mixtures, at low temperature, high pressure, and high H_2_S mole fraction.

More recently, Zheng et al. [68] probed the selective oxidation of H_2_S using the classical amino-modified NH_2_-MIL-53(Fe) and its parent analogue MIL-53(Fe), prepared by means of a simple hydrothermal method. Various characterization methods were carried out, such as x-ray diffraction (XRD), Brunauer–Emmett–Teller (BET), scanning electron microscopy (SEM), Fourier-transform infrared spectroscopy (FTIR), CO_2_ temperature-programmed desorption (CO_2_-TPD), and x-ray photoelectron spectroscopy (XPS), to provide an exegesis regarding the physicochemical properties of the solids. It was reported that the insertion of the amines decreased the activation energies for H_2_S oxidation and enriched the solid surface with moderate basic sites, thus, increasing the desulfurization performance. Specifically, the amino-functionalized material presented almost 100% sulfur selectivity in temperatures ranging from 130–160 °C, catalytically outperforming the commercial Fe_2_O_3_, MIL-53(Fe) and activated carbon that was evaluated against.

To further investigate the selective oxidation of H_2_S to sulfur, the same group prepared a porous MIL−100(Fe) with coordinatively unsaturated (CUS) Fe^2+^/Fe^3+^ sites (CUS-MIL−100(Fe)) [69]. It was reported that the functionalized materials exhibited high desulfurization performance and 100% sulfur selectivity at a temperature range of 100–190 °C and space velocity of 6400 h^−1^, outperforming both the commercial Fe_2_O_3_ and MIL−100(Fe). It is noteworthy that the H_2_S conversion over MIL−100(Fe) and CUS-MIL−100(Fe) remained stable at 100% in a continuous run of 100 h, while sulfur selectivity remained higher than 93.9% and 95.1%, respectively, indicating that these catalysts may be regarded as ideal candidates for practical applications. This enhanced catalytic performance of the CUS-MIL−100(Fe) can most likely be ascribed to the ordered pore structure of the MOF, which provides a chemically stable environment for the active pore sites, thereby hampering the development of potential side reactions.

## 3. H_2_S Capture via HKUST−1 (Hong King University of Science and Technology)

**Figure 7:**
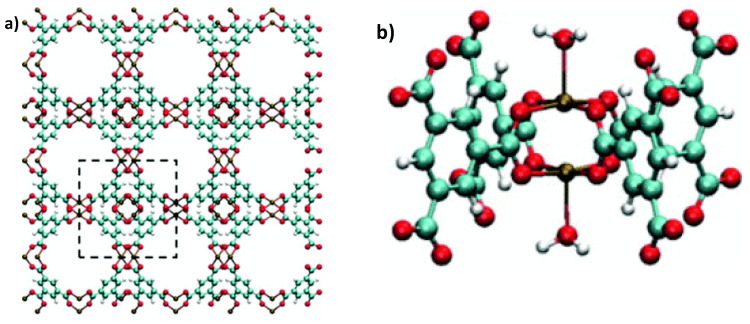
(**a**) Crystal structure of dehydrated Cu_3_(BTC)_2_ view along the [100] direction. Carbon, hydrogen, copper, and oxygen atoms are represented by the blue, white, brown, and red spheres. (**b**) The paddlewheel structure of a Cu dimer in Cu_3_(BTC)_2_ with water molecules coordinated to the metal centers. Reproduced with permission from Ref. [70]. Copyright 2010 The Journal of Chemical Physics.

Cu-BTC [Cu_3_(TMA)_2_(H_2_O)_3_]_n_ polymer, which also stands for MOF−199 or HKUST−1 (Hong King University of Science and Technology), consists of Cu nodes and organic linkers (benzene-tricarboxylate, BTC), with each Cu coordinated with four oxygen atoms and water molecules (Figure 7) [70]. The framework of this Cu-based material contains a bimodal pore size distribution, i.e., a large cage with 9 Å diameter and small pores with 3.5 Å diameter [71,72]. In addition, they exhibit a reasonable degree of thermal stability (up to 240 °C). In the recent past, various scientific works turned their attention to probing several aspects of H_2_S adsorption using this hybrid material [73,74,75,76,77,78,79,80,81,82,83,84,85,86,87,88,89].

Petit et al. [73] first investigated HKUST−1 and its composites with graphene oxide (GO) (5 to 46 wt.% graphene oxide) for H_2_S removal at ambient conditions using a flow of H_2_S (i.e., 1000 ppm, 250 ml min^−1^) diluted in moist air. The breakthrough capacities obtained denoted that the composite with 5 wt.% of GO exhibited the highest H_2_S uptake (199 mg g^−1^), while the capacities of HKUST−1 and GO were significantly lower (92 mg g^−1^ and 9 mg g^−1^, respectively). It was reported that H_2_O, which is present due to bed pre-humidification, does not prevent the H_2_S adsorption (2.7 mmol g^−1^). Conversely, it promotes its retention by means of dissolution in the water film. However, further H_2_S tests in absence of H_2_O would help to better elucidate the role of humidity. Following the H_2_S adsorption tests, the formation of CuS (black powder) was observed, illustrating the major disadvantage associated with the H_2_S adsorption on HKUST−1. Along these lines, the authors proposed that a possible H_2_S adsorption mechanism for HKUST−1 and the composite materials entails the substitution of H_2_O molecules coordinated to the Cu(II) metal centers by H_2_S. This mechanism hinted that the MOF structure is vulnerable to H_2_S exposure and may collapse. These observations were confirmed by XRD and IR spectroscopy. The authors also observed a decrement in pH during H_2_S adsorption, which is probably attributed to the acid nature of benzene-tricarboxylate linkers (no more coordinated to Cu). Overall, H_2_S capture in the presence of open metal sites can afflict the stability of the MOF framework. Results of this study show that MOFs based on metal clusters with open metal sites particularly confine the employment of these solids for H_2_S removal processes. However, the insertion of small amounts of GO can alleviate this limitation.

Pokhrel et at. [74] investigated the H_2_S adsorption on MOF−199 and MOF−199/GO. The authors argued that the GO did not favor the adsorption of H_2_S and attributed the observed capacity of MOF−199/GO was due to the presence of well dispersed crystals of the MOF. It was also reported that both physisorption and reactive adsorption occurred, owing to the unsaturated Cu sites in the MOF structure, which interact with H_2_S molecules. However, due to the fact that physisorption was the dominant capture mechanism, an increasing temperature led to favoring kinetics, but decreasing H_2_S capture. In the presence of moisture, the stability of MOF−199 and MOF−199/GO composites exhibited gradual degradation, suggesting that the unsaturated atoms of Cu in the metal center of MOF−199, having a coordination number of 4, could bind with H_2_O molecules via chemisorption.

In a more recent study by the same group [75] in-situ grown MOF−199 on GO, was doped with polyethyleneimine (PEI), and hybridized with GO along with functionalized GO for H_2_S capture. It was reported that, at ambient conditions, the pristine MOF−199 exhibited lower H_2_S adsorption capacity (0.5 mmol g^−1^) in comparison to that of the MOF−199/GO, which was pre-functionalized with low molecular weight PEI. In addition to the 80% improvement in H_2_S capture, the sorbent showed considerably enhanced sorption kinetics. Furthermore, MOF−199/GO hybrid sorbent exhibited 27% increase in H_2_S capacity (2.1 mmol g^−1^) in comparison to that of the pristine material by rising the temperature (150 °C). These GO-containing hybrid materials which are amenable to functionalizing both the GO support and MOF crystallites may be considered as promising alternatives for the removal of H_2_S.

Ebrahim et al. [76] studied composites consisting of HKUST−1 and S and N doped graphite oxides. The authors reported that the HKUST−1 composites exhibited improved adsorption performance in comparison to the parent MOFs, presumably owing to the insertion of polar groups and development of microporosity by linkages between the S and N groups of the modified GO and Cu centers of HKUST−1. In the presence of moisture, S doped GO/HKUST−1 and N doped GO/HKUST−1 showed increased H_2_S breakthrough capacities of 241 ± 6.44 mg g^−1^ and 125 ± 2.17 mg g^−1^, possibly due to the synergistic effect of the modified graphene phase. Synopsizing, H_2_O present, the adsorption capacities were improved in comparison to those in dry conditions, due to the dissolution and dissociation of H_2_S. The occurrence of acid-base reactions led to the formation of Cu/S salts instead of H_2_S, thus preventing the direct attack of H_2_S molecules on Cu centers of HKUST−1.

Zhang et al. [77] studied the effects of temperature and pressure on the performance of H_2_S adsorption in HKUST−1 using GCMC simulations. The interaction between adsorbate and adsorbent was further investigated through DFT calculations. The authors found that the H_2_S adsorption capacity on HKUST−1 increases with increasing pressure. When the pressure was higher than 0.4 Mpa, the adsorption tended to equilibrium. At low pressures, the capacity of the sorbent in capturing H_2_S molecules is strongly affected by the frameworks containing the binding sites of Cu dimers and benzene−1,3,5-tricarboxylic acid (trimeric acid). At high pressures, HKUST−1’s free volume also contributes to the adsorption performance of the MOF. Conversely, by increasing temperature, the H_2_S adsorption capacity of HKUST−1 decreases. This behavior is anticipated as the increased kinetic energy of H_2_S molecules compromised the effectiveness of the interactions between the framework atoms of HKUST−1 and guest H_2_S molecules. Consequently, residence time of H_2_S on the surface of HKUST−1 was shortened, resulting in poor adsorption performance. The interactions between H_2_S and organic linkers were weak (>−14.469 kJ/mol). It was reported that the most stable adsorption configuration for H_2_S to adsorb onto the organic linker, is the one with H_2_S located in the same plane as the benzene ring with hydrogen atoms of H_2_S nearby the oxygen atom of benzene−1,3,5-tricarboxylic acid. In addition, in the case that H_2_S adsorbs onto the Cu-Cu bridge, the binding energies of the modes with hydrogen placed inward of the Cu dimer are typically smaller in comparison to that where hydrogen is outward. The smallest binding energies (<−50 kJ/mol) were observed when the adsorption occurred on the top of the copper ion, probably owing to the trend of forming a saturated six-coordinated configuration.

**Figure 8:**
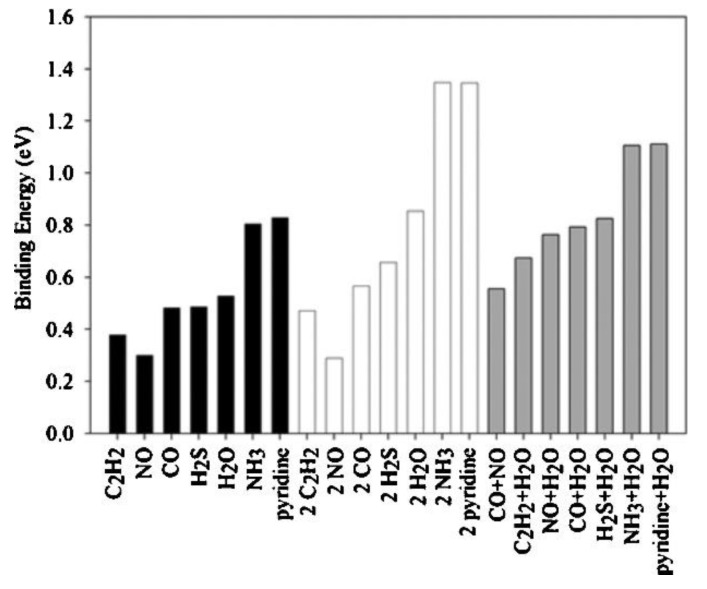
Binding energies of adsorbate molecules on a Cu dimer. Bars with black, white, and gray are for the binding modes of single molecules, two identical molecules, and combinations, respectively Reproduced with permission from Ref. [70]. Copyright 2010 The Journal of Chemical Physics.

A quite interesting work was conducted by Watanabe et al. [70] who tried to calculate the binding energies of several molecules (i.e., H_2_S, H_2_O, CO, NO, pyridine, C_2_H_2_, and NH_3_) on HKUST−1 using plane wave periodic density functional theory (DFT). H_2_S was found to exhibit a binding strength of 0.49 eV on Cu dimers, very close to that of H_2_O, which has shown a large affinity for the metal center of HKUST−1 (Figure 8). Employing GCMC simulations, at 27 °C and even at partial pressures less than 0.01 kPa, H_2_S was shown to adsorb at approximately 50% of active Cu sites, illustrating that HKUST−1 is a promising material for removing H_2_S from various industrial gases. It is worth mentioning that in the cases when two adsorbate molecules per dimer were adsorbed, the second molecule on a Cu dimer exhibited a lower binding energy. The effect of physisorption on the adsorption isotherm was not considered in this work.

Similar to the work discussed above [70], several other simulation studies reported that H_2_O molecules bind preferentially to the Cu atoms. Castillo et al. [78] noticed that H_2_O exhibits strong affinity for the metal centers in HKUST−1 compared to N_2_, CO_2_ or hydrocarbons. Kristof et al. [79] also corroborated that the H_2_O molecules interact more strongly with Cu atoms, whilst H_2_S molecules are accommodated at the center of the cages of the framework. Supronowicz et al. [80] observed that the interaction energy of H_2_O (−60.9 kJ mol^−1^) adsorption on HKUST−1 is higher than that of H_2_S (−52.25 kJ mol^−1^), hinting that H_2_O predominated. Gutiérrez-Sevillano et al. [81] developed H_2_S models to analyze whether a model that provides a precise dipole moment could predict an adsorption behavior (i.e., strong interaction of H_2_S with HKUST−1), in agreement with the experimental observation. The authors applied generic force fields for the material under consideration and found that the results appear in line with other relevant adsorption studies [52]. Moreover, DFT simulations showed that H_2_O, on the metal centers of HKUST−1, is energetically favored over the adsorption of H_2_S. Ab initio molecular dynamics of the molecules adsorbed on the model cluster show that the distance between H_2_S molecules and the metal center of HKUST−1 is longer by approximately 2.6 Å in comparison to H_2_O. Overall, the observations obtained from the simulation studies are in direct contradiction with the experimental results, which suggest the dominance of H_2_S physical adsorption on HKUST−1, thereby failing to explain that H_2_S converts Cu in HKUST−1 to black CuS in the presence of H_2_O. It is worth noticing that although the theoretical studies carried out so far consider the host-guest interactions, this is not the case for the thermodynamics governing the reaction, which are needed to explain the CuS formation. In concluding, experimental studies denote that H_2_S molecules show stronger affinity for Cu atoms in the center of HKUST−1, which make them capable of displacing H_2_O molecules.

Several works can be found in the literature have investigated the interaction between H_2_S molecules and HKUST−1 experimentally. For example, Ethiraj et al. [89] corroborated the structure collapse of HKUST−1 due to the formation of CuS. They reported that at higher equilibrium pressures (20–60 mbar), HKUST−1 exhibits a structural dilapidation possibly to owing to the conversion of Cu into CuS, while at lower equilibrium pressures (under 5 mbar) a stepwise structural distortion occurs. Alvarez et al. [83] suggested a similar adsorption mechanism for H_2_O molecules, concluding that the presence of H_2_O also compromises the stability of the HKUST−1.

Li et al. [84] studied the removal of H_2_S using HKUST−1 by activating the sample prior to H_2_S adsorption tests. The authors reported the positive effect of preheating treatment on HKUST−1due to the release of air and solvent molecules. In particular, the breakthrough capacity improved by 38% at 180 °C (optimum activation temperature) in comparison to the material that did not undergo activation and dropped by 10% at activation temperature of 200 °C. In addition, breakthrough tests demonstrated that the breakthrough capacity of HKUST−1 accreted with increasing temperature (from 30 to 80 °C). The aforementioned annotations highlighted the antagonistic adsorption between these two polar molecules (H_2_S/H_2_O) and that an increase in either adsorption temperature or activation temperature can lead to reducing the amount of adsorbed H_2_O on the sorbent, thus improving the H_2_S uptake. In the opposite direction, Peterson et al. [85], who also experimentally investigated the same system under dry (0% water) and wet conditions (80% H_2_O), reported that H_2_S adsorption capacity was not affected in both cases. Therefore, the contradicting results imply that there is room for better understanding the role of humidity in desulfurization using HKUST−1.

Zhang et al. [86] prepared a series of amine modified HKUST−1 via impregnation to remove H_2_S at ambient temperature. It was found that HKUST−1 could maintain its structure following modification with tertiary amine triethanolamine (TEA). However, this was not the case for amine mono-ethanolamine (MEA) and secondary amine diethanolamine (DEA) modification which led to the dilapidation of the HKUST−1’s structure, probably owing to the strong interactions occurred. Breakthrough results demonstrated that HKUST−1 functionalized by TEA exhibited higher H_2_S uptake (2.74 mmol g^−1^) in comparison to that of the parent sample (1.67 mmol g^−1^). Moreover, modification with DEA and MEA resulted in the decrease of H_2_S uptake to 0.58 mmol g^−1^ and 0.83 mmol g^−1^, respectively. Simulations showed that the binding energies of H_2_S adsorption on TEA/HKUST−1 were larger in comparison to those on pristine material, which presumably had a positive effect on H_2_S adsorption. The improved binding energy of H_2_S on the TEA/HKUST−1 can most likely be ascribed to the hydroxyl of TEA.

## 4. H_2_S Capture via Isoreticular Metal-Organic Frameworks (IRMOF-n)

**Figure 9:**
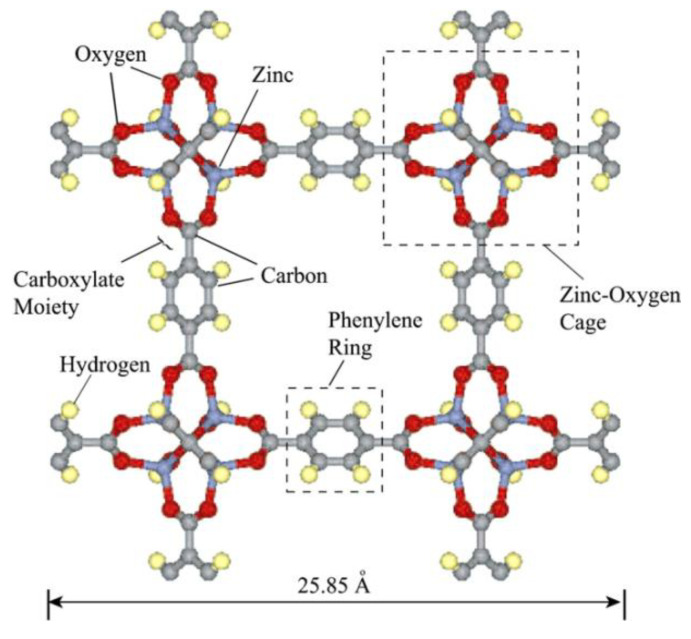
The cubic structure of MOF-5. The lattice constant at 27 °C is 25.85 Å. The diameter is 7.16 Å. Reproduced with permission from Ref. [88]. Copyright 2007 International Journal of Heat and Mass Transfer.

Isoreticular MOFs (IRMOF-n, where n = 1–16) based on a skeleton of Zn-based MOF were first prepared by Eddaoudi et al. [87]. An example of IRMOF−1 (also known as MOF-5) is presented in Figure 9 [88]; from the image it is clear that it has a stable cube-like structure with a regular, three-dimensional cubic lattice with BDC as edges and Zn_4_O cluster as vertexes 5.

In a theoretical study already discussed above, Gutiérrez-Sevillano et al. [81] investigated the adsorption of H_2_S on MOF-5. The authors reported lower heat of adsorption for MOF-5 (approximately −15 kJ mol^−1^) in comparison to that of HKUST−1 (approximately −30kJ mol^−1^) owing to wider pores of the MOF-5. Another interesting finding was that the energy of adsorption of H_2_O (−22.5 kJ mol^−1^) on MOF-5 was higher than that of H_2_S (−16.7 kJ mol^−1^), indicating that the presence of H_2_O molecules afflicts the adsorption of H_2_S.

Wang et al. [89] investigated the adsorptive removal of H_2_S at ambient temperature using IRMOF-3 (Zn_4_O(BDC-NH_2_)_3_), where BDC-NH_2_ represents 2-amino−1,4-benzenedicarboxylate. The authors probed the adsorption of dimethyl sulfide, ethyl mercaptan, and H_2_S using breakthrough adsorption curves at 30, 40, 50 and 60 °C. It was reported that increasing temperature results in decreased capture capacity in all cases. It is noteworthy that physical adsorption phenomena were observed from 30 to 60 °C. Moreover, the mechanism of H_2_S adsorption was studied by FTIR, XRD and XPS. The IR analysis showed a considerable change in the range of 3500–3300 cm^−1^, indicating a strong interaction through a hydrogen bond (H^δ+^ Ν^δ-^) between the H_2_S molecules and the -NH_2_ group of the MOF. Furthermore, the IR spectra demonstrated a new adsorption band at around 1620 cm^−1^, which was ascribed to H_2_O owing to a prospective chemical reaction (formation of ZnS). XPS results also corroborated the formation of ZnS, which is probably the product of the decomposition of the Zn_4_O-COO-_6_ cluster. Albeit IROMF-3 has metal center atoms that are sterically blocked by the linkers, the affinity of H_2_S for these Zn(II) metal centers, was quite higher in comparison to that of the -NH_2_ functional group. Afterwards, the authors investigated the effect of activation temperature. They found that the breakthrough capacity improved by 46% when the activation temperature was 150 °C in comparison to that of the sample that did not undergo activation and was significantly reduced (by 93%) at the higher activation energy of 200 °C. The positive effect of preheating treatment on HKUST−1’s adsorption capacity was also reported by Li et al. [84] and was rather attributed to the release of air and other solvent molecules. Increasing the adsorption temperature from 30 to 60 °C resulted in decreasing the breakthrough capacity to 19%.

**Figure 10:**
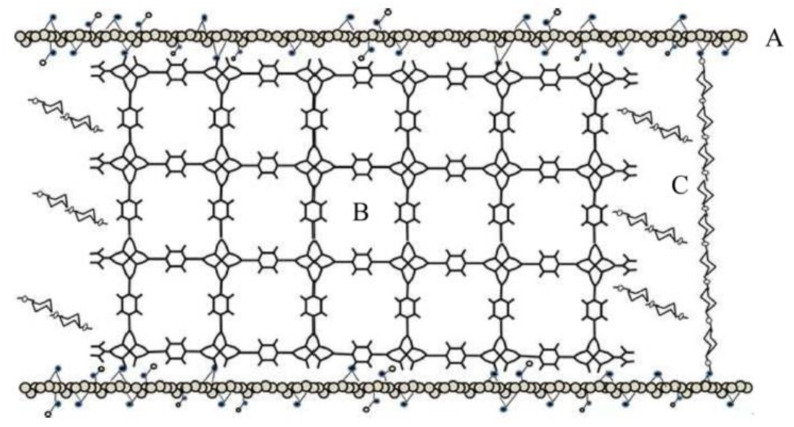
Schematic view of the glucose-promoted MOF-5/GO structure unit: (A) GO layer, (B) MOF-5, and (C) glucose polymer. Reproduced with permission from ref. [90]. Copyright 2012 Applied Materials & Interfaces.

Huang et al. [90] synthesized composites of Zn-based MOF (MOF-5) and GO in the presence of glucose for H_2_S removal (Figure 10). It was found that the glucose-promoted Zn-based sample exhibited increased H_2_S adsorption at 5.25% of GO loading, reaching a maximum capacity of 130.1 mg g^−1^. However, even though the loading of GO enhanced the dispersive force in the porous structure, when GO loading exceeded the optimum value of 5.25%, it resulted in the crystal distortion of the MOF-5. It was also claimed that the insertion of glucose can help maintain structural stability and prevent distortion.

## 5. H_2_S Capture via M-MOF-74

**Figure 11:**
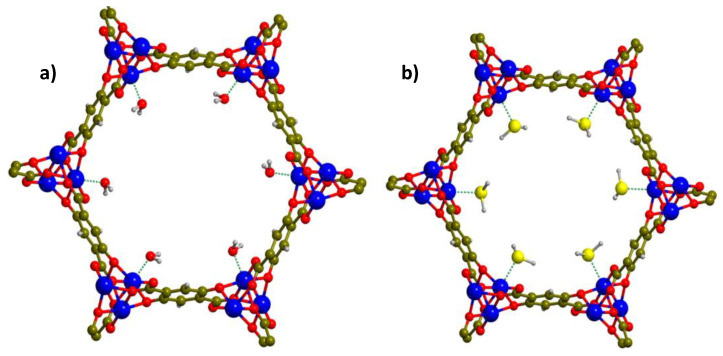
H_2_O molecules (**a**) and H_2_S molecules (**b**) as arranged in the Ni-MOF-74 channels. Metal, sulfur, oxygen, carbon, and hydrogen atoms are blue. Reproduced with permission from Ref. [92]. Copyright 2013 The Journal of Physical Chemistry C.

M-CPO-27 also known as M-MOF-74 [M_2_(2,5-dhbdc)(H_2_O)_2_], (2,5-dhbdc = 2,5-dihydroxyterephthalate M = Ni^2+^, Zn^2+^) was studied by Allan et al. [91] due to its strong affinity for H_2_S molecules. The authors showed that the amount of adsorbed H_2_S on Ni-MOF-74 was almost 6.4 mmol g^−1^ at room temperature and relative pressures (under 5 kPa). The highest H_2_S adsorption of 12 mmol g^−1^ was observed at 100 kPa and 25 °C. However, following regeneration, H_2_S adsorption was decreased in the second adsorption test, suggesting the irreversibility of H_2_S binding on the Ni sites. PXRD tests corroborated that structure retention following the H_2_S adsorption. Rietvelt refinements and Fourier difference plot analysis suggested that the principal binding site was the uncoordinated metal site, with Ni-S bond length of ~2.6 Å. In respect to Zn-MOF-74, it demonstrated significantly smaller H_2_S capture, probably owing to the reduced SSA of the sample, and the lower interaction energy of Zn in comparison to Ni with H_2_S. It was also reported that a relative amount of the stored H_2_S can be released by exposing Ni-MOF-74 (1.8 mmol g^−1^) and Zn-MOF-74 (0.5 mmol g^−1^) to moisture (i.e., 1.8 mmol g^−1^ and 0.5 mmol g^−1^, respectively, after 1 h) as the H_2_O molecules displace the H_2_S molecules at metal sites (Figure 11) [92]. In a nutshell, the interaction between Ni-MOF-74 and H_2_S was relatively weak to displace the oxygen atoms from the carboxylate linkers, in antithesis to the case of MOF−199, and generate nickel sulfide, but was quite strong to form coordination bond without degrading the MOF structure. The authors also showed that the presence of H_2_O, which strongly interacts with Ni-MOF-74 (~100 kJ mol^−1^), can compromise the H_2_S capture.

Chavan et al. [92] also probed H_2_S removal (relative pressures, 10 mbar) using Ni-MOF-74 and gave evidence for the formation of H_2_S adducts on almost 80% of the Ni sites. It was also stated that Ni-MOF-74 exhibited reversible behavior upon thermal activation at 200 °C for 12 h and that, following desorption, there was an increase in H_2_S uptake presumably owing to additional active sites produced via heat treatment.

## 6. H_2_S Capture via Universitetet I Oslo MOF (UiO-66)

**Figure 12:**
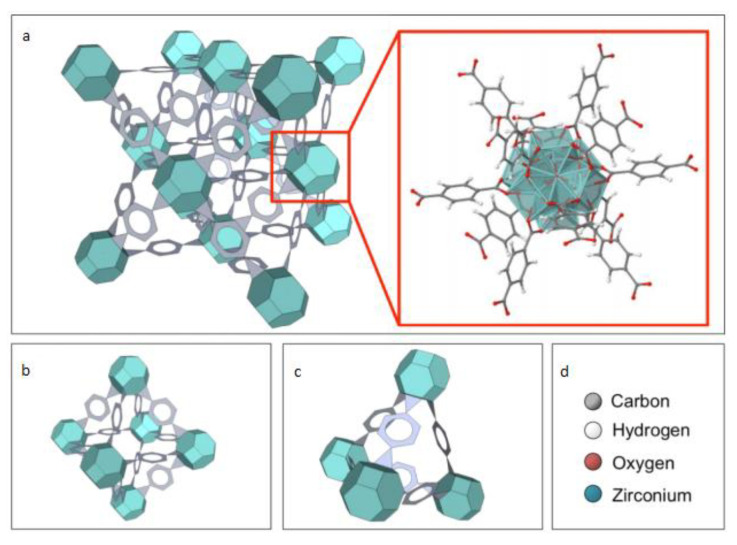
A representation of the UiO-66 structure. (**a**) The face-center-cubic UiO-66 structure composed of the metal node (aqua) and ligand (grey) with an atomic representation of the node and 12-connected terepthalic acid linkers from crystallographic data provided by Valenzano et al. in the Cambridge Crystallographic Data Centre, and generated from a visualizer using JSmol software at www.crystal.unito.it/vibs/uio66_hydro/ (**b**) The node and ligand structure composing the 12 Å UiO-66 cage. (**c**) The node and ligand structure composing the 7.5 Å cage. (**d**) The color scheme for the atomic representation. Reproduced with permission from Ref. [93]. Copyright 2019 Crystal Growth & Design.

UiO-66 (Universitetet I Oslo) is a MOF comprised of [Zr_6_O_4_(OH)_4_] clusters (octahedra) that are 12-fold connected with adjacent octahedra through BDC struts (linkers), resulting in a highly face centered cubic structure (Figure 12) [93] and one may find a few works in the literature that studied the removal of H_2_S using UiO-66 MOFs.

Li et al. [94] performed molecular simulations to investigate the adsorption performance of the pristine UiO-66(Zr) and its functionalized derivatives in capturing sulfur from binary gas mixtures. UiO-66-(COOH)_2_ and UiO-66-COOH exhibited the highest H_2_S adsorption capacity in comparison to that of other tested sorbents presumably due to their higher adsorption isosteric heats. The isosteric heat of adsorption at infinite dilution and radial distribution functions imply that the hydrophilic groups and polar H_2_S molecules strongly interact with one another, promoting H_2_S uptake.

**Figure 13:**
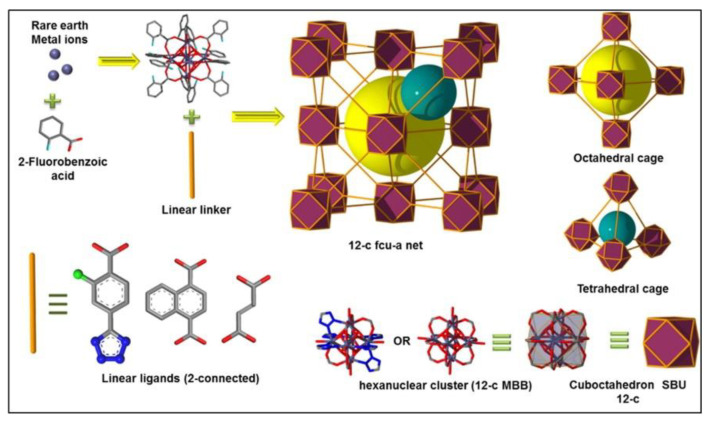
Assembly strategy for different analogs of fcu-MOFs with different linear linkers leading to isoreticular fcu-MOFs with different pore-aperture sizes. Reproduced with permission from Ref. [95]. Copyright 2017 Chemical Engineering Journal.

Bhatt et al. [95] revealed a series of isoreticular rare earth MOFs, namely RE-fcu-MOFs, and argued that these were highly stable and eclectic towards H_2_S molecules, with a face centered cubic (fcu) underlying topology (Figure 13). Having distinct pore-aperture sizes ranging from 4.7–6.4 Å and different functionalities these materials were studied for the removal of H_2_S from CO_2_ and CH_4_ containing gases using multiple cyclic adsorption breakthrough tests. In particular, the breakthrough tests were conducted on three isoreticular fcu-MOFs with different in length organic linkers (between 5 and 8.5 Å), using a simulated gas mixture with compounds typically found in natural gas streams CO_2_/H_2_S/CH_4_ (5%/5%/90%) under various adsorption-desorption conditions. Among the three selected Re-fcu-MOFs, the ones that were assembled by using the naphthalene moiety seemed to exhibit improved H_2_S uptake highlighting the crucial role of the pore system and functionality. Moreover, the fcu-MOF demonstrated decent H_2_S uptake with an increased H_2_S/CO_2_ selectivity, outperforming conventional materials (i.e., zeolites and activated carbons) in many aspects. It was also stated that the sufficient H_2_S/CO_2_ and H_2_S/CH_4_ selectivity of these materials can afford great opportunities to produce H_2_S, limiting the impurities of other compounds, such as CO_2_, to acceptable levels.

Recently, Daraee et al. [96] prepared TOUO-x nano composites (TiO_2_/UiO-66, x: 1, 3 and 5 wt.% of TiO_2_) and investigated their sulfur uptake performance. The results were then evaluated and compared with pristine UiO-66 at two different temperatures (30 and 50 °C) under space velocity of 30,000 h^−1^ and H_2_S concentration of 4000 ppm in the gas stream. Breakthrough results showed that rising temperature resulted in lower H_2_S uptake for the nanocomposites under consideration, hinting that the physical adsorption mechanism predominates. TOUO−1 outperformed the other tested materials (0.21 g S g^−1^) probably due to the higher SSA (1171 m^2^ g^−1^) in comparison to TOUO-3 (986 m^2^ g^−1^) and TOUO-5 (652 m^2^ g^−1^). Moreover, it is claimed that the insertion of metal active sites of titanium on UiO-66 contributed to the enhancement of the properties of the synthesized nano-composite adsorbents. Energy-dispersive X-ray spectroscopy (EDX) gave evidence for the presence of sulfur suggesting that there is a strong interaction between the sulfur molecules and the sorbent, probably due to chemisorption. However, the authors reported that following two cycles of H_2_S adsorption tests, the capacity of TOUO−1 was almost fully regenerated (its capacity decreased by approximately 3–5%), which somehow contradicts the fundamental knowledge that chemisorption results in irreversible structure transformation.

Trying to investigate the antagonistic adsorption between CO_2_ and H_2_S, Huang et al. [97] synthesized core-shell-structure H_2_S imprinted polymers (PMo_12_@UiO-66@H_2_S-MIPs) based on the surface of UiO-66 modified by phosphomolybdic acid hydrate. Initially, it was reported that using H_2_O as substitution template for H_2_S can overcome issues associated with the toxic and instable nature of H_2_S. It was shown that PMo_12_@UiO-66@H_2_S-MIPs exhibited increased H_2_S uptake (24.05 mg g^−1^) in comparison to that of the carrier PMo_12_@UiO-66, indicating that the desulfurization performance of the latter was further enhanced by the H_2_S imprinted polymers. Moreover, PMo_12_@UiO-66@H_2_S-MIPs showed high H_2_S uptake at room temperature even in the presence of humidity and had decent separation towards H_2_S/CO_2_. A noteworthy finding was that air purge regenerated PMo_12_@UiO-66@H_2_S-MIPs at 180 °C and O_3_ treatment and ambient temperature demonstrated stable desulfurization ability after six cycles. The authors observed the transformation of H_2_S into sulfur following adsorption, as well as that the PMo_12_ was the redox agent in the desulfurization process.

## 7. H_2_S Capture via Zeolitic Imidazolate Frameworks (ZIFs) 

One of the major issues that impedes the practical applications of MOFs is their poor hydrothermal stability. A case in point is MOF-5, whose structure starts to collapse irreversibly after only 10 min of exposure to humidity, even under low pressure and temperature [98]. Due to the hydrophilic property of some MOFs, they strongly interact with H_2_O molecules. Therefore, even infinitesimal amounts of H_2_O, which is usually formed during reactions or contained on humid air, can attack, and cleave the coordination bonds, leading to the destruction of frameworks. The hydrophilic property of MOFs also hinders the access of hydrophobic organic substrates to the active sites, compromising the catalytic activity of some reactions [99,100].

To take advantage of the stability of zeolites, combined with the diverse structure and tunable functionality of MOFs, zeolitic imidazolate frameworks (ZIFs), which are classified as a MOF sub-class, have also been used for adsorption applications. ZIFs, are zeolite-like structures which are composed of transition metal ions that replace aluminum or silica atoms and maintain the topology of a zeolitic material. In addition, the organic linkers displace the oxygen atoms in the lattice of the zeolite. ZIF-8, has received extensive attention, due to the fact that it exhibits high thermal stability (up to 550 °C), relatively high surface area (1630 m^2^ g^−1^) and notable chemical resistance to boiling organic solvents and alkaline H_2_O [101]. Ethiraj et al. [82] reported higher stability of ZIF-8 over HKUST−1. However, due to fact that most ZIFs are Zn based, there is the widely known issue of ZnS formation, resulting in the collapse of the structure of the material.

## 8. Additional Works

Peng et al. [102] performed GCMC simulations, wherein the dispersive interactions between atoms are delineated by directly applying the universal force field (UFF) from Rappe et al. [103], for zeolites, porous carbons and MOFs to remove H_2_S and CO_2_ from ternary mixtures (i.e., CH_4_, CO_2_, H_2_S). It was reported that by considering only the desulfurization capacity, zeolites are superior to porous carbons and MOFs, whilst for concurrent desulfurization, MOFs outperformed the other two types of sorbents. MOFs also experienced easier regeneration in comparison to zeolites, since the adsorbed molecules can be easily and quickly released by increasing temperature. Moreover, by increasing the amount of H_2_S in the bulk phase, the selectivities of MOFs decrease with a slower rate, in comparison to those of zeolites, which shows that the MOFs are better candidates for the desulfurization of gases with impurities in high concentrations. Along these lines, socMOF and indium-based rho-zMOF, which is representative of zeolite-like MOFs (zMOFs) with a topology of rho-zeolite, have been found to exhibit high selectivity (100 and 170, respectively) and capacity (1.8 and 2.6 mmol g^−1^).

**Figure 14:**
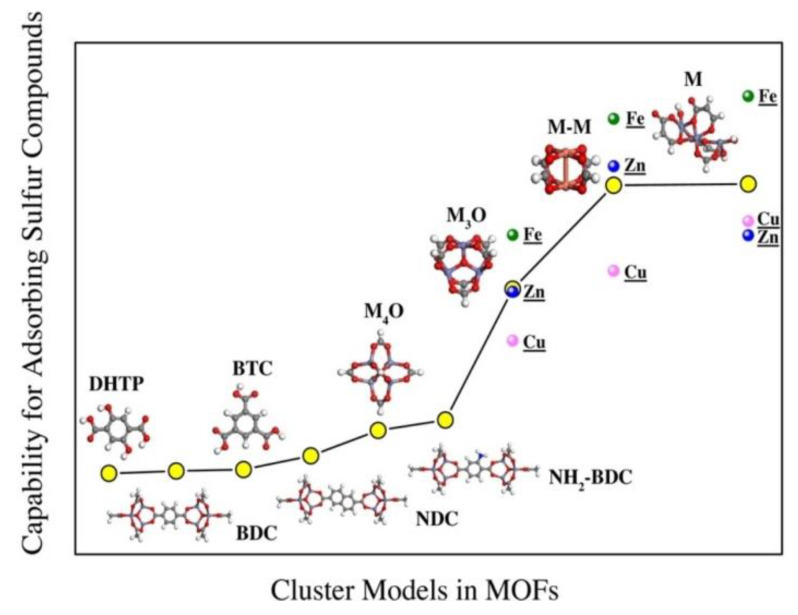
Adsorption of sulfur compounds under the effect of different organic linkers (NH2-BDC, BDC, and NDC), metal centers structures (M, M-M, and M_3_O) and metal ions (Zn, Cu, and Fe). Reproduced with permission from Ref. [104]. Copyright 2016 Applied Surface Science.

In an attempt to gain better insights into the desulfurization performance of MOFs, Chen et al. [104] probed the contribution of each fragment of these hybrid sorbents (metal center structures, metal ions, organic linker) to the adsorption of sulfur compounds (H_2_S, dimethyl sulfide (CH_3_SCH_3_), ethyl mercaptan (CH_3_CH_2_SH)) using DFT. The authors reported that the capture of sulfur compounds on organic linkers, NH_2_-BDC, BDC (1,4-benzenedicarboxylate), and NDC (dimethyl 2,6-naphthalenedicarboxylate) is realized through physical adsorption. NH_2_-BDC exhibited the strongest binding strength owing to the functional amino-group, which enhances the polarity in the linker and thereby improves its interaction with sulfur. Moreover, metal centers with structure M and M-M in HKUST−1 and MOF-74 showed strong binding strength. The chemical interaction between the metal centers of HKUST−1 and MOF-74 and the adsorbed sulfur compounds was evidenced by the fact that the sorbents exhibited their weakest binding energy in −51.8 kJ mol^−1^. Meanwhile, it was revealed that MOFs containing Fe demonstrated stronger binding with sulfur compounds in comparison to those containing Cu and Zn. However, the strong binding of H_2_S to Fe also implies a risk of iron sulfide formation. Overall, the adsorption strength of MOFs with sulfur species followed the order of: MOFs with coordinatively unsaturated sites > MOFs with NH_2_-BDC linker > MOFs with saturated metal center > MOFs with the organic linkers without substituent group (Figure 14).

Liu et al. [105] probed, both experimentally and theoretically, the adsorption mechanism and stability of eleven different MOFs when challenged with H_2_S separation. The H_2_S breakthrough tests and the experimental N_2_ isotherms were conducted at 25 and −196 °C, respectively. Most of these MOF-based materials exhibited on-off increased capacity and selectivity to H_2_S/CO_2_. In particular, Mg-MOF-74, MIL−101(Cr), UiO-66, ZIF-8, and Ce-BTC experienced complete reversible physical sorption, while irreversible H_2_S chemisorption was observed for Cu-BTC, Zn-MOF-74 and MOF-5 (albeit, leading to high H_2_S uptake and H_2_S/CO_2_ selectivity). Nevertheless, as is usually the case, the presence of end products (e.g., ZnS, CuS) may afflict the structure of the sorbents and limit their further exploitation. Regarding HKUST−1, Cu-BDC(ted)_0.5_, Zn-MOF-74, MIL−100(Fe) gel, and MOF-5, and despite the fact that they experienced the same irreversible chemical adsorption, their structures remained stable. It was also observed that in the case of MIL−100(Fe) the redox reaction between Fe and H_2_S (i.e., H_2_S reduces Fe(III) to Fe(II)) can result in the formation of octasulfur (S8). The authors tried to shed light into the possible H_2_S adsorption mechanisms, using DFT, molecular dynamics and dynamic separation experiments, for Mg-MOF-74, MIL−101(Cr), UiO-66, ZIF-8, and Ce-BTC. It was reported that the chemical composition of the MOF holds a key role in the H_2_S adsorption. For instance, in MIL−101(Cr) and Mg-MOF-74 with open metal sites, there are electrostatic interactions between the unsaturated coordination sites and the H_2_S molecules (Μ^δ+^·SH_2_^δ−^), resulting in the physisorption of H_2_S. On the other hand, in MOFs with no evident access to the open metal sites, H_2_S capture was realized through CH·π hydrogen bond, where H_2_S is the donor and the organic linker (aromatic rings) are the acceptors. In respect to the Ce-BTC sorbent, which affords access to open metal sites, high band gap (1.16 eV) in the highest occupied molecular orbital (HOMO)-lowest unoccupied molecular orbital (LUMO) energy levels of the p orbital from H_2_S and f orbital from Ce, leads to the physisorption of the target molecule (H_2_S). Eventually, Cu-BDC(ted), which contains a metal oxide cluster paddlewheel-type [Cu_2_(O_2_C-)], experienced an H_2_S/amino group interaction that can stabilize the structure and produce amine-hydrosulfide adduct.

Eddaoui et al. [106] introduced fluorinated MOFs in polymer membrane with a view to remove acid gases (H_2_S and CO_2_) from natural gas. Namely, they employed NbOFFIVE−1-Ni (KAUST-7) and AlFFIVE−1-Ni (KAUST-8) with a general formula of [M_1_(M_2_F_x_)(pyrazine)_2_]_n_ where M_1_ is Ni, and M_2_F_x_ are the pillars [NbOF_5_]^2−^ and [AlF_5_]^2−^, respectively. The results of adsorption isotherms for H_2_S, CO_2_, and CH_4_ at 35 °C showed increased CO_2_/CH_4_ selectivity and improved capacity in capturing H_2_S molecules for both fluorinated materials, as well as adequate stability following H_2_S exposure. Thereby, selective transport of these gases via a membrane process was studied. The authors used 6FDA-DAM polyimide polymer matrices for the synthesis of mixed-matrix membrane (MMM), and they showed that incorporating these fluorinated MOFs in these polymer matrices resulted in enhanced diffusion for the acid gases (CO_2_/H_2_S) in comparison to that of CH_4_. In this regard, KAUST-7 and KAUST-8 exhibited improved molecular diffusion, through the membranes presumably due to the well-defined channels that provided.

In a very promising work, Belmabkhout et al. [107] investigated different fluorinated MOF-based materials for the removal of H_2_S and CO_2_ with a view to upgrade biogas, natural gas, and refinery-off-gas. It was reported that the fluorinated MOF, AlFFIVE−1-Ni allowed the synchronous and equally selective removal of these acid gases (i.e., H_2_S and CO_2_) from streams with high CH_4_ concentrations in a single adsorption step. On the other hand, SIFSIX-2-Ni-I exhibited preferential binding only towards H_2_S molecules. A significant finding was that AlFFIVE−1-Ni demonstrated 100% regenerating efficiency at mild conditions (105 °C). Overall, simultaneous removal of both H_2_S and CO_2_ can be attained by means of the integrated favorable sites for H_2_S and CO_2_ uptake in a confined pore system.

**Figure 15:**
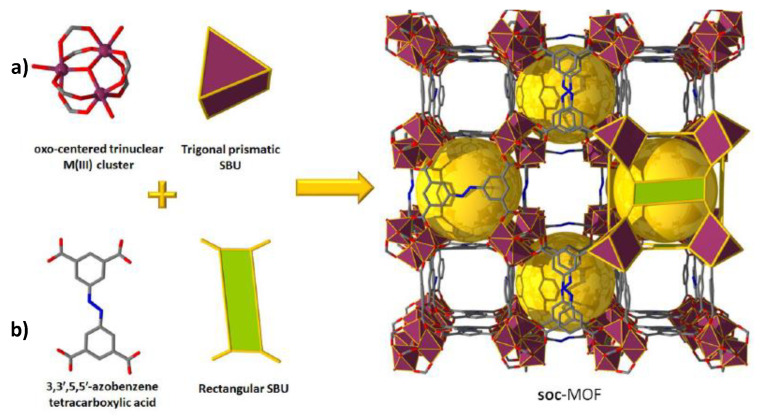
Structure of the soc-MOF: (**a**) polyhedral representation of the μ3-oxygen-centered trinuclear metal carboxylate clusters ([M_3_O(O_2_C-)_6_], where M = In^3+^, Fe^3+^, Ga^3+^ and Al^3+^, which can be viewed as a 6-connected node having a trigonal-prismatic geometry); (**b**) representation of the organic ligand (ABTC, which is shown as a 4-connected rectangular-planar geometry); crystal structure of the cuboidal cage-type soc-MOF (M, plum; C, gray; N, light blue; and O, red; the cavity space is indicated by yellow vdW spheres; hydrogen atoms, Cl^−^ and NO^3-^ ions are omitted for clarity). Reproduced with permission from Ref. [108]. Copyright 2017 Journal of Materials Chemistry A.

Belmabkhout et al. [108] also studied In^3+^-, Fe^3+^-, Ga^3+^-, and the newly isolated Al^3+^-based isostructural square octahedral (soq)-MOFs with a view to produce high-quality hydrocarbons (CH_4_, C_3_H_8_ and n-C_4_H_10_, and olefins), from H_2_S-containing gas streams (Figure 15). It was reported that only Al^3+^- and Ga^3+^-based soc-samples could exhibit structural stability during the interaction with H_2_S molecules. Pure-component adsorption isotherms were measured for H_2_S, CO_2_, and CH_4_ in Ga-soc-MOF at ambient temperature and, differently from CO_2_ and CH_4_, H_2_S exhibited steep adsorption. The authors also conducted breakthrough experiments for a ternary rich in CH_4_ mixture (i.e., 5% of H_2_S, 5% of CO_2_, 90% of CH_4_) for six adsorption-desorption cycles under various conditions in a cyclic fashion, which is relevant to temperature and vacuum swing regeneration. Complete recyclability of the MOF was accomplished at 160 °C under He flow. H_2_S breakthrough time of 40 min, which was considerably longer in comparison to CH_4_ (0 min) and CO_2_ (5 min) breakthrough times, illustrates very high H_2_S/CH_4_ and H_2_S/CO_2_ selectivities. It was also claimed that Al-soc-MOF exhibited reasonable degree of structural stability for temperatures up to 300 °C and relative humidity (RH) as high as 95%. Nevertheless, the H_2_O stability of the Ga-soc-MOF was not mentioned.

Lee et al. [109] investigated, both experimentally and theoretically, the chemisorption of H_2_S using tree different MOFs (MOF−199, MOF-5, and UiO-66-NH_2_), two covalent-organic polymers (COPs: CBAP−1 (EDA) and CBAP−1 (DETA)), and two commercial sorbents (Carbopack-X and activated carbon (AC)) in an attempt to evaluate the adsorptive performance of these samples (inlet stream partial H_2_S pressure: 10 ppm in 1 bar of N_2_, temperature = 25 °C). Despite the fact that the adsorption capacity at 100% breakthrough volume (BTV) has been mentioned as the most common fashion to assess the performance of a sorbent, the authors employed the 10% BTV for that purpose, which has been claimed as a more practical performance metric [110,111]. Most adsorbents exhibit a steep rise in breakthrough levels following the initiatory adsorption event (≤10% breakthrough). That said, the actual potential of an adsorbent may be evaluated more accurately with the earlier attainment of breakthrough level (e.g., 10%). The authors noted that MOF−199 exhibited a noticeable advantage in terms of the adsorptive performances determined at BTV10 over the other tested samples. In particular, the materials followed the order MOF−199 > MOF-5 > AC > UiO-66-NH_2_ > CBAP−1 (EDA) > CBAP−1 (DETA) > Carbopack-X. Considering the isotherm modeling, it was shown that MOF−199 can effectively adsorb H_2_S molecules by means of formation of irreversible chemical bonds with Cu-Cu site bridge. A significant divergence in the partition coefficients between the experimental and theoretical data was also noted, probably because the crystal lattice of MOF−199 is more stable under real-world conditions. Finally, the authors hypothesized that Mg/Cu-based dual metal site MOFs could be a way to surpass the present limitations (i.e., collapsed framework due to chemisorption) in H_2_S adsorption. At this point it should be stressed that, to the best of our knowledge, evaluating capacity at 10% BTV is not necessarily panacea. Capacity at full breakthrough volume gives the equilibrium capacity and has a thermodynamic meaning, while capacity at 10% BTV attempts to simulate the impact of the mass transfer zone in a real separation process. Nevertheless, considering the MOFs are unused or fully regenerated in a powdered form, then the mass transfer zone in the lab breakthrough experiments is in any case not representative.

Li et al. [112] conducted a computational study on composite materials based on Cu-TDPAT MOF and ionic liquids. The MOF was comprised of metal oxide clusters in a paddlewheel-type form [Cu_2_(O_2_C-)] linked to a 2.4.6-tris(3,5-dicarboxylphenylamino)−1,3,5-triazine (TDPAT) linker. Structurally, it has a three-dimensional network with tree different pores, as well as shortened tetrahedral and octahedral cages with sizes of 9.1, 12.0, 17.2 Å, respectively. In respect to ionic liquids, the authors altered the anions (i.e., bis[(trifluoromethyl)-sulfonyl]imide [TF_2_N]ˉ, tetrafluoroborate [BF_4_]ˉ, chloride [Cl]ˉ, with a view to selectively adsorb H_2_S in a binary mixture of H_2_S/CH_4_, but they maintained the same cation [BMIM]^+^ (1-n-butyl-3-methylimidazolium). Results showed that the anions, exhibited an affinity towards copper atoms while cations were accommodated nearby N atom from triazine. Regarding the [BF_4_]ˉ, it was the only anion that located close to the N atom from amino group. Radial distribution functions (RDF) demonstrated distances between anions and copper atoms in the order of [Cl]ˉ < [BF_4_]ˉ < [PF_6_]ˉ < [TF_2_N]ˉ, being in consonance with the trend of ionic radius. Consequently, it can be concluded that the size of the anions holds a key role in the accommodation of ionic liquids into the pores of MOF. Moreover, [Cl]ˉ anion was only found on the tetrahedral cavity, probably since it is sterically blocked, whilst the other anions occupied all pores. In addition, the isosteric heats of adsorption were measured at infinite dilution (Q°_st_) for CH_4_ and H_2_S for both Cu-TDPAT and Cu-TDPAT-composites. Furthermore, computational observations from simulated adsorption selectivities displayed that the increased selectivity of H_2_S over CH_4_ was ascribed to the fact that H_2_S, contrary to CH_4_, is polar. In conclusion, this type of materials based on ionic liquids could be an alternative for H_2_S capture as they have the potential to protect the metal cluster with open metal sites, preventing the structural disintegration by nucleophilic onset of H_2_S molecules. However, the regenerability of these materials under the appropriate conditions was not investigated in this work.

Sánchez-González et al. [113] mentioned the use of Mg-CUK−1 MOF (CUK = Cambridge University-KRICT), which is built from a 2,4-pyridinecarboxylate (2,4-pdc) linker and Mg(II) octahedral centers, linked to infinite chains of [Mg_3_(μ_3_-OH)]^5+^ clusters by μ_3_-OH groups placed inside the pore walls, for the removal of acidic gases, such as H_2_S, in the presence of relative humidity. In particular, to elucidate the impact of humidity, the authors attempted to characterize the adsorption/desorption properties of H_2_O. According to the H_2_O adsorption isotherms, the highest H_2_O uptake was almost 19.1 mmol g^−1^ and was not affected after several adsorption/desorption cycles. Afterwards, they tried to delineate the H_2_S sequestration performance on activated Mg-CUK−1 MOF following several adsorption/desorption cycles. Results showed that the H_2_S capture increased with increasing the H_2_S concentration. Namely, at 6% volume the H_2_S capture reached at 1.41 mmol g^−1^, while at 15% volume was equal to 1.41 mmol g^−1^. Furthermore, with a view to corroborate that this solid can maintain its crystallinity after H_2_S exposure, the authors conducted five adsorption-desorption cycles combined with PXRD analyses. It was found that the Mg-based material maintained its capacity in capturing H_2_S between the cycles, suggesting that the structure was not afflicted during the process. Finally, GCMC results demonstrated that H_2_S molecules were mainly bonded via hydrogen bonding (μ_3_-OH···SH_2_). In this case, the hydroxyl group, which is located at the wall of the MOF, is the proton donor. At higher H_2_S loadings the aforementioned interaction becomes weaker (−23.3 kJ mol^−1^), which explains the easiness of the Mg-CUK−1’s regeneration between adsorption/desorption cycles.

Zárate et al. [114] investigated H_2_S capture in an Al(III)-based MOF, named MIL-53(Al)-TDC (TDC = 2,5-thiophenedicarboxylate) by performing both experimental and theoretical tests. Structurally, this material contains [AlO_4_-trans-(μ-OH)_2_] octahedra in which Al(III) center is coordinated by six oxygen atoms from four different TDC linkers and two hydroxyl (μ-OH) groups. The capture performance of MIL-53(Al)-TDC was firstly evaluated by breakthrough tests conducted at 30 °C, 1 bar and 5% of H_2_S volume, the results of which suggested increased H_2_S capture of 18.13 mmol g^−1^. Similar results (18.1 mmol g^−1^) were obtained by kinetic gravimetric H_2_S tests. Following H_2_S exposure, the MOF seems to retain its framework, which is something that PXRD analysis corroborates. In addition, thermogravimetric analysis (TGA) showed the complete removal of H_2_S molecules at the relatively low temperature of 65 °C. Afterwards, five adsorption/desorption cycles were carried out (5% of H_2_S volume), exhibiting a basically constant H_2_S regeneration capacity (~18.5 mmol g^−1^) for MIL-53(Al)-TDC. This reversibility indicated weak interactions between H_2_S molecules and the pores of MOFs. To corroborate these results the authors conducted DRIFT tests and GCMC simulations. The computational study demonstrated that H_2_S molecules interact inside the pores via hydrogen bonding (μ-OH·SH_2_). Furthermore, H_2_S molecules attract one another (adsorption band at ~3491 cm^−1^) through dipole–dipole interactions, which constitute the main reason of increased H_2_S capture. As expected, H_2_S molecules also interact with the organic linker (thiophene). Finally, the low H_2_S adsorption enthalpy (−23.2 kJ mol^−1^), justifies the weak nature of these interactions.

Generally, the literature shows that MOFs with metal complexes μ_x_-OH (μ_3_-OH for Mg-CUK−1, μ_2_-OH for MIL-47(V), MIL−125(Ti), and MIL-53(Cr)) exhibit respectable H_2_S reversibility at ambient temperature. In respect to MIL-53(Al)-TDC (μ-OH), although it demonstrates considerably high H_2_S uptake, it bears a major shortcoming, which is the high-temperature reactivation process (activated at 200 °C for 4 h under a flow of dry N_2_ gas) compared to the room-temperature regeneration conditions.

The last work presented herein was carried out by Mohideen et al. [115], who reported the synthesis and construction of kag-MOF−1 [Zn(HCN_4_)_2_·(H_2_O)]·1,4(H_2_O), which is crystallized in a hexagonal system, comprised of two crystallographically independent Zn cations, in an octahedral environment. The authors showed that kag-MOF−1 due to its high localized charge density, which is attributed to the presence of relatively inexpensive tetrazolate organic building units, and one-dimensional channels with small in size pore-apertures, has great potential in removing acid gases such as H_2_S.

Synopsizing, Table 1 presents information regarding the textural properties (specific surface area, pore size, and pore volume), experimental conditions used and H_2_S adsorption capacity (mmol g^−1^) of selected MOFs mentioned herein. The adsorption mechanisms and the factors governing selectivity are key when dealing with H_2_S capture on MOFs. H_2_S can interact with the open metals forming sulfides (e.g., CuS, ZnS), which results in the collapse of their structure, as in the case of HKUST−1 (MOF−199) and ZIF-8. Moreover, these sorbents, display low H_2_S adsorption performance in comparison to the MIL family.

In this regard, large pore MIL−100(Cr), MIL−101(Cr) and MIL−101(Cr)-4F demonstrate high SSA, rigid structure and considerably high H_2_S; a drawback is the poor reversibility of these materials. On the other hand, despite the fact that small pore MIL-47(V) and MIL-53(Al, Cr) show reduced capturing performance in comparison to that of the large pore MILs, they are more flexible, and their structure can be partially recovered. Furthermore, MIL-53(Al)-TDC show considerable high H_2_S uptake at low temperature (30 °C) and pressure (0.1 MPa) as well as noteworthy reversibility and decent moisture stability. However, high reactivation conditions are required.

When evaluated against MILs, UiO-66 also illustrate a relatively low H_2_S adsorption capacity, though, but it seems to effectively resist to metal sulfide formation. The incorporation of -NH_2_ can further improve its H_2_S capturing performance, but it negatively affects its reversibility. Another MOF with high H_2_S uptake is Ni-MOF-74 which has also the advantage of a stable structure when exposed to H_2_S molecules. Notwithstanding, in the presence of water, the H_2_S adsorption capacity decreases. A major shortcoming is also the high reactivation conditions. Conversely, Mg-CUK−1 can be reactivated under low conditions, but its capacity in capturing H_2_S is limited (3.03 mmol g^−1^).

**Figure 16:**
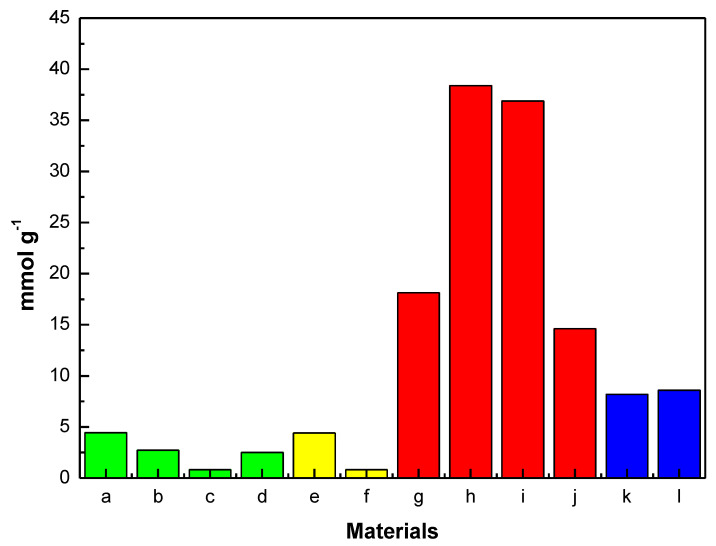
Adsorption capacities of several materials towards H_2_S molecules (green: zeolites, yellow: metal oxides, red: MOFs, blue: activated carbons): (**a**) Zeolite 13X [64], (**b**) Zeolite 4A [64], (**c**) PEI(50)/MCM-48 [116], (**d**) SAPO-43 [117], (**e**) iron sponge [118], (**f**) Cu_0.5_Zn_0.5_/Al_2_O_3_ [119], (**g**) MIL-53(Al)-TDC [114], (**h**) MIL−101(Cr) [49], (**i**) MIL−101(Cr)-4F(1%) [66], (**j**) MIL-47(V) [49], (**k**) AC (Desorex K43-Na) [120], and (**l**) AC (Desorex K43-BG) [120].

Finally, to reinforce the comparative hypostasis of this work, Figure 16 shows the H_2_S capture capacities of common adsorbents (zeolites, metal oxides and activated carbons) and some of the most promising MOFs mentioned in the literature. It is evident that MOFs can be considered as one of the most interesting and promising candidates for the removal of toxic and corrosive H_2_S molecules as they outperform the classic porous sorbents, owing to their enhanced chemical properties and functionality of the linkers and cavity dimensions.

## 9. Mechanisms of H_2_S Capture Using MOFs

The adsorption mechanisms and the factors governing selectivity are key when dealing with H_2_S capture on MOFs [121]. H_2_S can chemisorb on the metals by forming sulfides, it can interact with coordinatively unsaturated metal sites by Lewis acid-base interactions, it can interact with basic linkers (e.g., amines) by acid-base interactions, or simply be physisorbed via dipolar interactions.

Chemisorption occurs when there is evidence of electron transfer between adsorbents and adsorbates during the adsorption process. Sometimes, the formation of a strong and often irreversible metal–sulfur bond can cleave other coordination bonds between the metal centers and linkers, leading to a structural dilapidation of the MOF’s framework, as in the case of H_2_S capture in HKUST−1 modified with mono-ethanolamine and diethanolamine [86]. Consequently, it is necessary to modulate the host-guest binding interactions between the target molecules (e.g., H_2_S) and MOFs.

In physisorption, the adsorptive interaction is largely ascribed to noncovalent interaction (i.e., electrostatic interactions and Van Der Waals), which is highly associated with the distance between adsorbents and adsorbates. Moreover, the reversibility of an adsorption process is pertinent to the occurrence of noncovalent bonding between the functionalized linkers and H_2_S molecules [29].

A number of computational (density functional theory (DFT) methods and grand canonical Monte Carlo (GCMC) simulations) and experimental studies have focused on the optimization of the interactions between MOFs and H_2_S to gain a deeper understanding in terms of H_2_S adsorption mechanisms [70,78,79,80,81].

## 10. Connection between Functions and Structural Features of MOFs

In this final section, we focus on the H_2_S capture properties of the MOFs and the connection between the removal abilities and the structures. We provide a summary of the unique structural features of MOFs while we emphasize the relationship between the structure and physical properties including pore profile, SSA, and thermal and mechanical stability.

As mentioned above, the main reason MOFs exhibit multiple functions is their diverse crystalline structures with tunable pore sizes. MOFs are constructed from metal nodes that are coordinated by rigid and aromatic organic linkers which are mostly carboxylic acid or nitrogen-containing linkers. Modifiable surfaces and tunable pores for specific applications (e.g., gas separation) can be achieved by judiciously selecting the appropriate metal clusters and organic linkers. The defined MOF structures offer the opportunity of investigating the relationship between the structure and physical properties, which is crucial for the design of MOFs with desirable functionality.

The functional sites of MOFs can be chemically produced by the pore surfaces. For instance, amine functional groups that can recognize small molecules should be incorporated into the organic ligands. However, it is critical to carefully select the metals and organic linkers as they bear different properties and may be incompatible compromising the performance of the material. Furthermore, the pore size of MOFs ranges between microporous to microporous, which facilitates the coexistence of diverse molecules such as metal complexes, polymers, metal atoms etc. These guest species can considerably enhance the functionality of MOFs (multiple functions). In a theoretical work, Gutiérrez-Sevillano et al. [81] studied the adsorption of H_2_S on MOF-5 and reported lower heat of adsorption for MOF-5 (approximately −15 kJ mol^−1^) in comparison to that of HKUST−1 (approximately −30 kJ mol^−1^) due to wider pores of the MOF-5.

Even though different MOF structures have been reported so far, they often suffer from weak stability, namely acid base stability, H_2_O stability, and thermal stability, hindering their use in industrial applications. For example, Alvarez et al. [83] concluded that the presence of H_2_O, which usually coexists with H_2_S in gas streams, compromises the HKUST−1’s stability since the metal-coordinated linkers are replaced by H_2_O or OHˉ. Generally, since many MOFs bear hydrophilic properties, they strongly interact with H_2_O, which means that even low amounts of H_2_O can break the coordination bonds, resulting in structural degradation. Consequently, a method to enhance the stability of the material is to strengthen the coordination bonds between metal nodes and organic linker. In this regard, H_2_O-stable MOFs, such as MIL−101 family, ZIFs, and zirconium-based carboxylates among others, have been extensively probed for desulfurization in the presence of moisture. Along these lines, new schemes by constructing hydrophobic surfaces or interfaces to improve H_2_O-stability have also been developed recently [122,123,124,125]. A noteworthy work was conducted by Bae et al. who treated MOF−199 with O_2_ plasma and maintained the porosity of the MOFs following hours of H_2_O exposure, thus improving the surface polarity of the MOF [126].

An even more challenging task is to construct MOFs with decent acid/base stability. Essentially, hard/soft acid/base (HSAB) theory states that soft Lewis acids react faster and form stronger bonds with soft Lewis bases, whereas hard Lewis acids react faster and form stronger bonds with hard Lewis bases [75,127]. However, considerable progress has been made in developing MOFs with acid/base stability. A widespread method is to combine high oxidation state metal ions with carboxylate linkers (i.e., hard acid and hard base) to generate strong bonds with resistance to chemical attacks. Another common method to eliminate H_2_S via an acid/base reaction is the use of different amines for modifying MOFs and to eventually improve H_2_S uptake, owing to their available nitrogen sites and high affinity for acid gases. The amine loading aims to simultaneously increase the SSA and affinity towards H_2_S molecules without hampering diffusion and accessibility of H_2_S molecules in the internal pores and surface of the MOFs. That said, controlled grafting at molecular precision is critical to prevent the blockage of the pores, and therefore forestall the access of the functional moieties inside the pore volume, while the amine groups are attached to the open metal sites [128]. However, this method is not efficient in competitive CO_2_/H_2_S adsorption since both gases are electron acceptors (Lewis acids) and they have similar effect with amine.

The matter of thermal stability of MOFs is also of paramount importance as high temperatures are also required for different applications (e.g., activation of the sorbent prior to H_2_S adsorption). This parameter is typically determined by the node-ligand bond strength and the number of ligands connected to each metal node. High valence metal ions such as Al^3+^, Cr^3+^, Zr^4+^ usually generate materials that can resist to high temperatures. Typically, most MOFs are stable up to 350–400 °C [28].

Another important factor for practical applications is mechanical stability of MOFs under vacuum or pressure. Even though high SSA and porosity of MOFs can be beneficial for an adsorption process, they also make MOFs less stable from a mechanical perspective. Permanent and highly ordered porosity and high SSAs provide the basis for the multiple functionalities of MOFs. While high SSA is typically beneficial to gas adsorption processes, the pore volume of MOFs is least as important since it affords the physical upper limit of the amount of the stored gas. Microporous MOFs with pore diameter lower than 2 nm have demonstrated increased adsorption capacities for various gases including H_2_S. In addition, high micropore volumes and large SSAs are desirable for many application, as narrow pores inhibit the insertion of large unwanted impurities. The mesoporous MOFs MIL−100(Cr) and MIL−101(Cr) have also been investigated for desulfurization processes with interesting results [49,50]. Different methods have arisen that are efficient at constructing and tuning the pores of MOFs. Trying to elongate the length of linkers is a common and efficient strategy to achieve this task. In principle, the linear extension of organic linkers can improve pore size of MOFs in each network. However, sometimes, the openness of the structure can lead to interpenetration, thus preventing the space from being accessible [32].

## 11. Conclusions

Bearing in mind the toxic and corrosive nature of H_2_S, the need for efficacious removal processes is obvious. Adsorption appears to predominate over the other gas separation methods mostly due to its cost-effectiveness. Searching through the available literature, it becomes apparent that MOFs can afford significant advantages in gas selectivity and separation, as well as increased H_2_S uptake in comparison to other porous materials.

However, the ability of MOFs in capturing H_2_S molecules can be compromised by the formation of strong and sometimes irreversible bonds. The chief cornerstone to surpass this limitation is to regulate the host–guest binding interactions between MOFs and H_2_S molecules.

Reversibility following H_2_S exposure can be attained through noncovalent bonding between functionalized linkers and H_2_S. A deeper understanding of the preferred H_2_S adsorption sites is key to regulate and enhance the H_2_S capture.

Structural characteristics of the material seem to hold the lion’s share in successfully completing these tasks. To illustrate this point, the structure of MOFs with open metal sites, such as HKUST−1, IRMOF-3 and MIL-53(Fe) disintegrates when challenged with H_2_S molecules, forming metal sulfides. An effective strategy to overcome this limitation is the use of MOF-composites as in the case of MOF-GO, where graphene oxide is used as a support. Nevertheless, these composite materials exhibit poor H_2_S adsorption capacity.

On the other hand, when the strength of interactions between the open metal sites and H_2_S molecules is moderate, and H_2_S cannot displace the oxygen atoms from the carboxylate linkers (i.e., Ni-CPO), a coordination bond is formed without breaking the material’s structure, which affords an alternative to remove H_2_S via a combination of physical and chemical phenomena. This option offers the opportunity of a relatively reversible process.

Moreover, the functionalization of the MOF’s surface can result in increased H_2_S uptake. For example, the insertion of 1% of a fluorinated linker in MIL−101(Cr)-4F(1%) allows for enhanced H_2_S capture. Although noticeable efforts have been made in studying the adsorption capacity of H_2_S using MOFs, there is a clear need for gaining a deeper understanding in terms of their thermal conductivities (lack of studies in the literature) and specific heats in order to design more stable adsorption beds, experiencing high exothermicity. Simply put, the exothermic nature of adsorption means that sharp rises in temperature can negatively affect the bed stability in the absence of sufficient heat transfer. Future studies should also take advantage of the benefits afforded by reticular chemistry for the development of stable porous MOFs, appropriate for sweetening applications.

It is also worth noticing that numerous simulation studies have played a crucial role in the elucidation of the mechanism of the H_2_S capture in MOFs in the presence of H_2_O. Nevertheless, the modeling predictions in regards to the effect of H_2_O on the adsorption of H_2_S are in direct contradiction with the experimental results on the grounds that, while they take into account the host–guest interactions, this is not the case for the thermodynamics governing the reaction, which are required to explain the formation of sulfides.

In conclusion, although considerable advances have been made in terms of MOFs structural features in the past two decades, the development of MOF structures with enhanced H_2_S selectivity, higher H_2_S uptake, regenerability, long-term stability, and lower operational and fabrication cost remains a challenge that needs to be addressed before the commercialization of MOFs.

## Figures and Tables

**Table 1:** SSA, experimental conditions and H_2_S removal characteristics of selected MOFs.

Material-Code	SSA (m^2^ g^−1^)	Pore Size (Å)	Pore Volume (cm^3^ g^−1^)	Conditions Tested	H_2_S Capture (mmol g^−1^)	Source
MIL-53(Cr)	1946	~11	-	1.6 MPa, 30 °C	13.12	[49]
MIL-53(Al)	1103	~11	-		11.77	
MIL-53(Fe)	-	~11	-		8.53	
MIL-47(V)	930	~11	-	2 MPa, 30 °C	14.60	
MIL−100(Cr)	2000	4.8 × 5.8 and 12.5 × 12.5	-		16.70	
MIL−101(Cr)	2916	4.8 × 5.8 and 12.5 × 12.5	-		38.40	
MIL−125-(Ti)-NH_2_	1245	-	0.54	0.01 MPa, 30 °C	2.00	[53]
MIL−101-HNO_3_	3841	-	1.72	3.5 MPa, 20 °C	27.16	[64]
MIL−101(Cr)-4F(1%)	2176	-	1.19	0.2 MPa, 30 °C	36.90	[66]
MOF−199	1387	~11.5	0.45	Ambient conditions	2.70	[73]
MOF−199-GO	989	-	0.52		5.80	
MOF−199-GO/PEI	-	-	-	0.1 MPa, 150 °C	2.10	[75]
MOF−199-TEA	186	-	-	0.1 MPa, 25 °C	2.74	[86]
Ni-MOF-74	1545	-	-	0.1 MPa, 25 °C	12.00	[91]
UiO-66	1322	-	-	0.001 MPa, 25 °C	0.23	[105]
UiO-66-NH_2_	1097	-	-		0.91	
ZIF-8	1602	-	-		0.05	
Mg-MOF-74	1244	-	-		0.24	
Ce-BTC	930	-	-		0.13	
Zn-MOF-74	920	-	-		1.64	
MOF-5	2250	-	-		1.11	
MIL−100(Fe) gel	816	-	-		0.90	
Mg-CUK−1	604	-	0.22	0.1 MPa, 30 °C	3.03	[113]
MIL-53(Al)-TDC	1400	-	-	0.1 MPa, 30 °C	18.13	[114]
